# Comparison of the global prevalence and trend of human intestinal carriage of ESBL-producing *Escherichia coli* between healthcare and community settings: a systematic review and meta-analysis

**DOI:** 10.1093/jacamr/dlac048

**Published:** 2022-06-02

**Authors:** Yihienew M. Bezabih, Alemayehu Bezabih, Michel Dion, Eric Batard, Samson Teka, Abiy Obole, Noah Dessalegn, Alelegn Enyew, Anna Roujeinikova, Endalkachew Alamneh, Corinne Mirkazemi, Gregory M. Peterson, Woldesellassie M. Bezabhe

**Affiliations:** 1 Arsi University College of Health Sciences, University Road, Asella, ET 0193, Ethiopia; 2 Department of Internal Medicine, WellStar Atlanta Medical Center, Atlanta, GA, USA; 3 Bahir Dar University, Bahir Dar, Ethiopia; 4 University of Nantes, Microbiotas Hosts Antibiotics and bacterial Resistances Laboratory, Nantes, France; 5 CHU Nantes, Emergency Department, Nantes, France; 6 Marshall University School of Medicine, Huntington, WV, USA; 7 Department of Microbiology, Monash University, Clayton, Victoria 3800, Australia; 8 School of Pharmacy and Pharmacology, University of Tasmania, Hobart, Australia

## Abstract

**Objectives:**

The widespread intestinal carriage of ESBL-producing *Escherichia coli* (ESBL *E. coli*) among both patients and healthy individuals is alarming. However, the global prevalence and trend of this MDR bacterium in healthcare settings remains undetermined. To address this knowledge gap, we performed a comparative meta-analysis of the prevalence in community and healthcare settings.

**Methods:**

Our systematic review included 133 articles published between 1 January 2000 and 22 April 2021 and indexed in PubMed, EMBASE or Google Scholar. A random-effects meta-analysis was performed to obtain the global pooled prevalence (community and healthcare settings). Subgroup meta-analyses were performed by grouping studies using the WHO regions and 5 year intervals of the study period.

**Results:**

We found that 21.1% (95% CI, 19.1%–23.2%) of inpatients in healthcare settings and 17.6% (95% CI, 15.3%–19.8%) of healthy individuals worldwide carried ESBL *E. coli* in their intestine. The global carriage rate in healthcare settings increased 3-fold from 7% (95% CI, 3.7%–10.3%) in 2001–05 to 25.7% (95% CI, 19.5%–32.0%) in 2016–20, whereas in community settings it increased 10-fold from 2.6% (95% CI, 1.2%–4.0%) to 26.4% (95% CI, 17.0%–35.9%) over the same period.

**Conclusions:**

The global and regional human intestinal ESBL *E. coli* carriage is increasing in both community and healthcare settings. Carriage rates were generally higher in healthcare than in community settings. Key relevant health organizations should perform surveillance and implement preventive measures to address the spread of ESBL *E. coli* in both settings.

## Introduction

The widespread intestinal carriage of ESBL-producing *Escherichia coli* (ESBL *E. coli*) among patients and healthy individuals is alarming.^1^ This is because ESBL *E. coli* can cause MDR infections that are difficult to treat. In healthcare settings it can cause serious hospital-acquired infections that have a 3-fold increased mortality compared with infections caused by non-drug-resistant *E. coli* strains.^2^ In a community setting, it can lead to community-acquired MDR infections, such as recurrent urinary tract infections (UTIs), with an increased risk of morbidity.^3–6^

In many studies, human faecal ESBL *E. coli* carriage prevalence was higher in hospital settings than in the community.^7–9^ This could be related to the use of antibiotics, which is an independent risk factor for ESBL *E. coli* faecal colonization.^10–14^ In addition, antibiotic-mediated dysbiosis and loss of gut colonization resistance could facilitate the transmission (person-to-person contact, food and water ingestion, environmental contact) and acquisition of ESBL *E. coli* in the hospital setting.

In our recent systematic review and meta-analysis that evaluated the prevalence of faecal carriage of ESBL *E. coli* among healthy individuals, we found a global community prevalence of 16.5%, showing a dramatic 8-fold rise over the last two decades.^1^ Our findings were in line with the earlier studies that showed that the pooled prevalence of ESBL Enterobacteriaceae in the community (more than 90% of which is accounted for by ESBL *E. coli*^10^) was 14% in 2016.^15^

However, the global prevalence and trend over time of this MDR bacterium in healthcare settings remains undetermined. In addition, no prior study has compared global carriage rates among patients and healthy individuals. Hence, in this meta-analysis, we explored the global prevalence of human ESBL *E. coli* faecal carriage in healthcare settings and compared it with the values found in the community. Furthermore, as the acquisition of intestinal ESBL *E. coli* carriage could rise with increasing hospital stay,^9,14^ we compared carriage rates among patients with varying duration of hospitalization and in individuals living in nursing care facilities.

## Methods

This meta-analysis was conducted following the preferred reporting items for systematic reviews and meta-analyses (PRISMA) 2020 checklist^16^ (Table S1, available as Supplementary data at *JAC-AMR* Online).

### Data sources and search terms

The data sources were articles published between 1 January 2000 and 22 April 2021 and obtained by a systematic search in PubMed, EMBASE and Google Scholar. Four groups of search terms were used to find articles that determined the intestinal carriage rate of ESBL *E. coli* among patients admitted to healthcare settings (including individuals living in a nursing care facility): (i) *Escherichia coli* OR *E. coli*; (ii) extended spectrum β-lactamase OR ESBL; (iii) faecal OR faeces OR stool OR intestinal OR gastrointestinal tract; (iv) hospital OR hospital-acquired or admitted OR inpatient OR nursing home. These groups of search terms were then connected by the Boolean operator ‘AND’ to find papers that contained the terms anywhere in the article. Similarly, articles for the community setting were found using the above search terms except for the substitution of ‘community OR community-acquired’ in place of ‘hospital OR hospital-acquired or admitted OR inpatient OR nursing home’. For the healthcare setting, we retrieved a total of 1239 articles (328, 631 and 280 articles indexed in PubMed, EMBASE and Google Scholar, respectively) for further screening. For the community setting, we obtained a total of 590 articles (129, 181 and 280 articles indexed in PubMed, EMBASE and Google Scholar, respectively). The reference lists of the included papers were also checked to identify relevant studies. Screening of the articles by their titles and abstracts was performed by two authors (Y.M.B. and W.M.B.), and one author (A.B.) helped in reaching a consensus with any discrepancies.

### Study selection: inclusion and exclusion criteria

Studies that determined the prevalence of ESBL *E. coli* carriage among patients (healthcare settings) or healthy individuals (community setting) of any age were eligible. Patients in the healthcare settings were defined as individuals who were kept at the emergency department or admitted to wards, ICU or a nursing care facility for any kind of treatment or care. Healthy individuals were defined as asymptomatic individuals living in the community, including those who visited a health facility for a routine wellness check-up, vaccination, antenatal care, pre-international travel screening, or transrectal biopsy screening for prostate cancer. In this manuscript, the terms ‘healthy individuals’ and ‘asymptomatic individuals’ were interchangeably used to describe individuals living in the community setting, and do not imply the absence of comorbidities or symptoms of illness. We used the term ‘stool sample’ for faecal samples collected either by the routine stool or rectal sampling.

We made four categories of study subjects by the duration of contact with a healthcare setting at the time of stool sampling: (i) healthy individuals (in the community); (ii) admitted <48 h; (iii) admitted ≥48 h; and (iv) living in nursing care facilities. We considered healthy individuals as having zero time of contact in a healthcare setting, whereas individuals living in nursing care were considered to have an indefinite time of contact. We categorized patients as ‘admitted <48 h’ if the faecal carriage rate in these patients was determined using a stool sample that was taken at admission or within 48 h of hospital admission. Participants were categorized as ‘admitted ≥48 h’ if the prevalence was determined from a stool sample taken after 48 h of admission, or from serial culturing from admission till discharge, or if the time of sampling was not specified.

**Figure 1.**
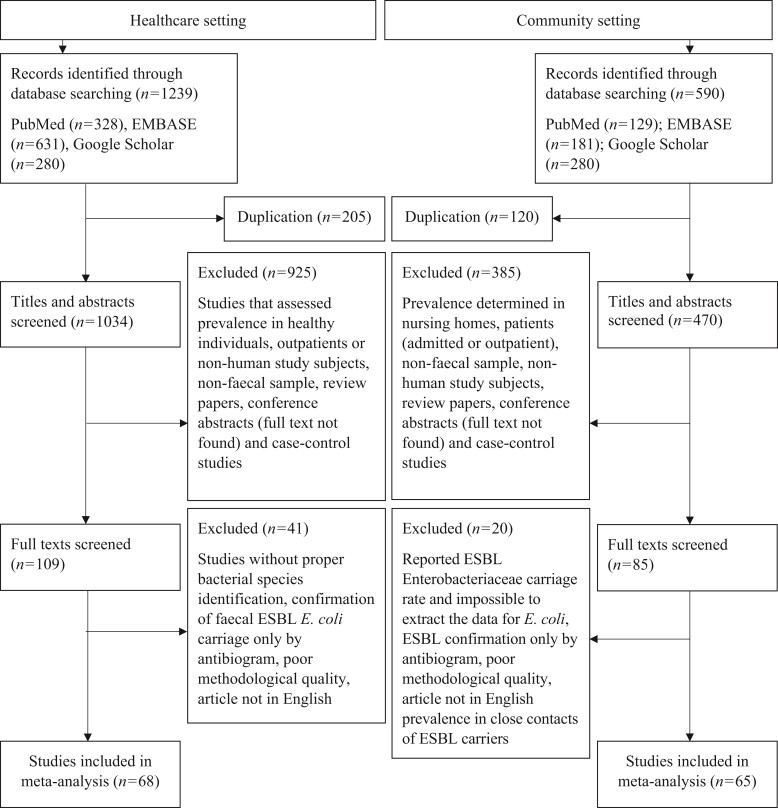
Selection of articles for the meta-analysis. Note: studies reporting the prevalence in both the community and healthcare settings were not discarded.

We included original articles written in English and excluded case-control studies, reviews and conference abstracts. Studies that reported the prevalence of faecal ESBL *E. coli* among outpatients with recurrent UTI were excluded due to disproportionately high intestinal carriage rates of ESBL *E. coli* in such patients.^17,18^ We have also excluded studies that determined ESBL *E. coli* carriage rates in returning travellers from countries with a high prevalence or among household contacts of colonized individuals; those that involved non-human study subjects or analysed non-faecal samples; and studies that measured the prevalence of faecal carriage of ESBL Enterobacteriaceae, but without bacterial species identification. Furthermore, we only included studies that used at least the double-disc synergy test (DDST) or PCR to confirm ESBL production and excluded those studies that relied on the routine antibiogram to detect resistance to cephalosporins. A flow chart showing the selection of articles pertaining to ESBL *E. coli* prevalence in both community and healthcare settings is shown in Figure 1.

### Data extraction and quality control

The prevalence of the human intestinal carriage of ESBL *E. coli* (the main outcome of interest) was calculated by dividing the total number of ESBL *E. coli*-positive individuals by the total number of individuals screened in each study. We also extracted data on the year of study, study design, nature of study participants, method of ESBL confirmation and study location (country and WHO region) (Table S2).

The quality of each study was assessed using the quality assessment tool for observational cohort and cross-sectional studies developed by the National Heart, Lung, and Blood Institute of the NIH^19^ (Table S3).

### Data analysis

A random-effects meta-analysis using the DerSimonian and Laird method^20^ was performed to obtain the global pooled prevalence for each setting (community and healthcare). Subgroup meta-analyses were performed by grouping studies using the WHO regions^21^ and 5 year intervals of the study period. For studies with a duration of more than 1 year (e.g. 2013–14), the approximate mean (2014) was taken as the year of study. The global trend of faecal ESBL *E. coli* carriage was demonstrated in two ways: (i) linear regression analysis and (ii) by using a pooled prevalence after articles grouped by 5 year intervals of the study period. The Freeman–Tukey arcsine methodology^22^ was used to stabilize the variance of raw proportions, and no studies with 0% or 100% proportions were excluded.^23^ The measure of heterogeneity was the I^2^ statistic.^20^ Probability values less than 0.05 at a 95% CI were considered significant. Egger’s regression test was used to assess the presence of publication bias.^24^ The OpenMeta (Analyst) software was used to perform the meta-analysis.^25^ GraphPad Prism (version 8.0.2, San Diego, CA, USA) was used to create linear regression plots and bar graphs.

## Results

### Study characteristics and quality assessment

**Figure 2.**
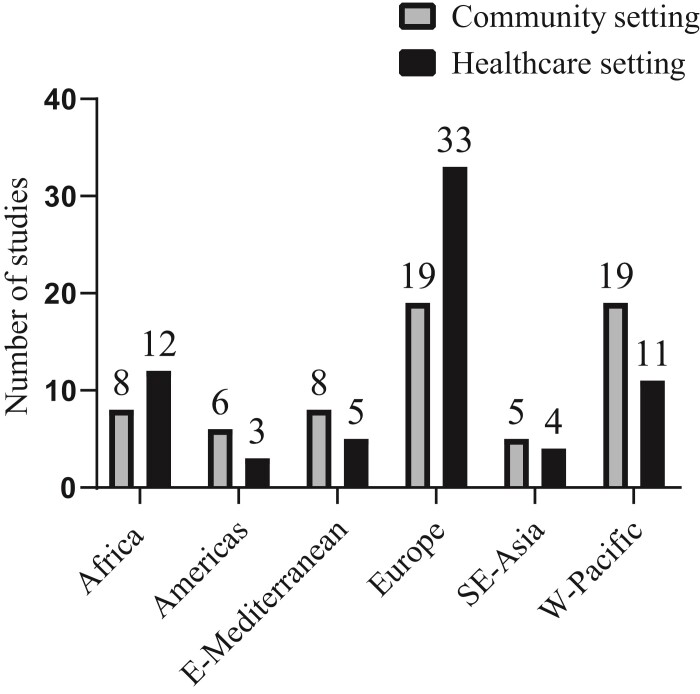
Number of studies included by WHO region and study setting. The absolute numbers of participants (ESBL *E. coli* positive/total screened) in the community settings were (by region): Africa (270/1786), America (109/1242), South-East Asia (494/1502), Europe (887/15168), Eastern Mediterranean (479/2084) and West Pacific (1713/8851). The absolute numbers of participants (ESBL *E. coli* positive/total screened) in the healthcare settings were (by region): Africa (682/2206), America (107/2236), South-East Asia (304/1494), Europe (3370/32464), Eastern Mediterranean (218/711) and West Pacific (1040/3574).

A total of 133 articles covering 73 318 participants were included in the meta-analysis (Figure 2, Figure S1). This included non-duplicate stool samples from 30 633 healthy individuals (65 articles in community settings) and 42 685 inpatients (68 articles in healthcare settings) (Figures 1 and 2, Figure S1). The majority of the studies in both the community (19/65, 29.2%) and healthcare (33/68, 48.5%) settings were from Europe (Figure 2). The study period of the included studies ranged from 2003 to 2018 for the community setting and 2002 to 2019 for the healthcare setting. All included studies were either cohort or cross-sectional studies with a fair to good quality (Table S2).

### Comparison of global and regional prevalence in faecal ESBL E. coli carriage between the community and healthcare settings

**Figure 3.**
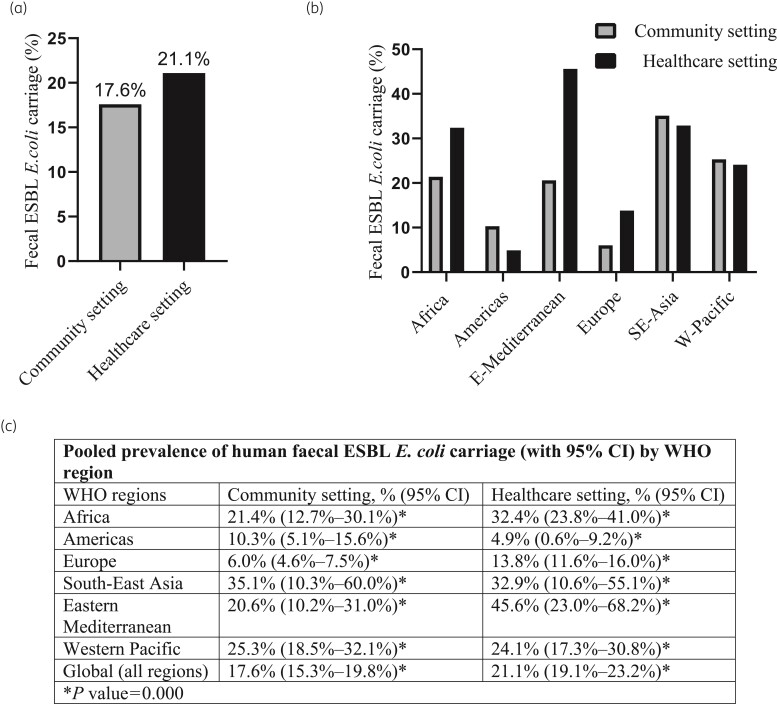
The global and regional prevalence of human intestinal ESBL *E. coli* carriage. (a) The global pooled prevalence of faecal ESBL *E. coli* carriage in community and healthcare settings (forest plot in Figure S1). (b) Regional pooled prevalence of faecal ESBL *E. coli* carriage in the six WHO regions (forest plot in Figures S2 and S3). (c) A summary of global and regional human intestinal ESBL *E. coli* carriage with 95% CI as obtained from forest plots in Figures S1–S3. *P* values are the *P* values for heterogeneity.

Overall, the global and regional carriage rates generally appeared higher in healthcare than in community settings, although the 95% CIs overlapped in most of our analyses (Figure 3). Globally, the cumulative (2000–21) pooled prevalence of intestinal ESBL *E. coli* carriage in healthcare settings was 21.1% (95% CI, 19.1%–23.2%) compared with 17.6% (95% CI, 15.3%–19.8%) in the community settings (Figure 3a, Figure S1).

In the community setting, by WHO region, the highest carriage rates occurred in South-East Asia (35.1%, 95% CI, 10.3%–60.0%), followed by the West Pacific (25.3%, 95% CI, 18.5%–32.1%), Africa (21.4%, 95% CI, 12.7%–30.1%) and Eastern Mediterranean (20.6%, 95% CI, 10.2%–31.0%). Europe (6.0%, 95% CI, 4.6%–7.5%) and the Americas (10.3%, 95% CI, 5.1%–15.6%) had the lowest reported ESBL *E. coli* colonization in the community (Figure 3b and c, Figures S2 and S3).

In contrast, in healthcare settings, the highest carriage rate was found in the Eastern Mediterranean (45.6%, 95% CI, 23.0%–68.2%), followed by South-East Asia (32.9%, 95% CI, 10.6%–55.1%), Africa (32.4%, 95% CI, 23.8%–41.0%) and the West Pacific (24.1%, 95% CI, 17.3%–30.8%), whereas the lowest colonization rate was seen in the Americas (4.9%, 95% CI, 0.6%–9.2%) and Europe (13.8%, 95% CI, 11.6%–16.0%) (Figure 3b and c, Figures S2 and S3).

### Global and regional trends in prevalence of human intestinal ESBL E. coli carriage

**Figure 4.**
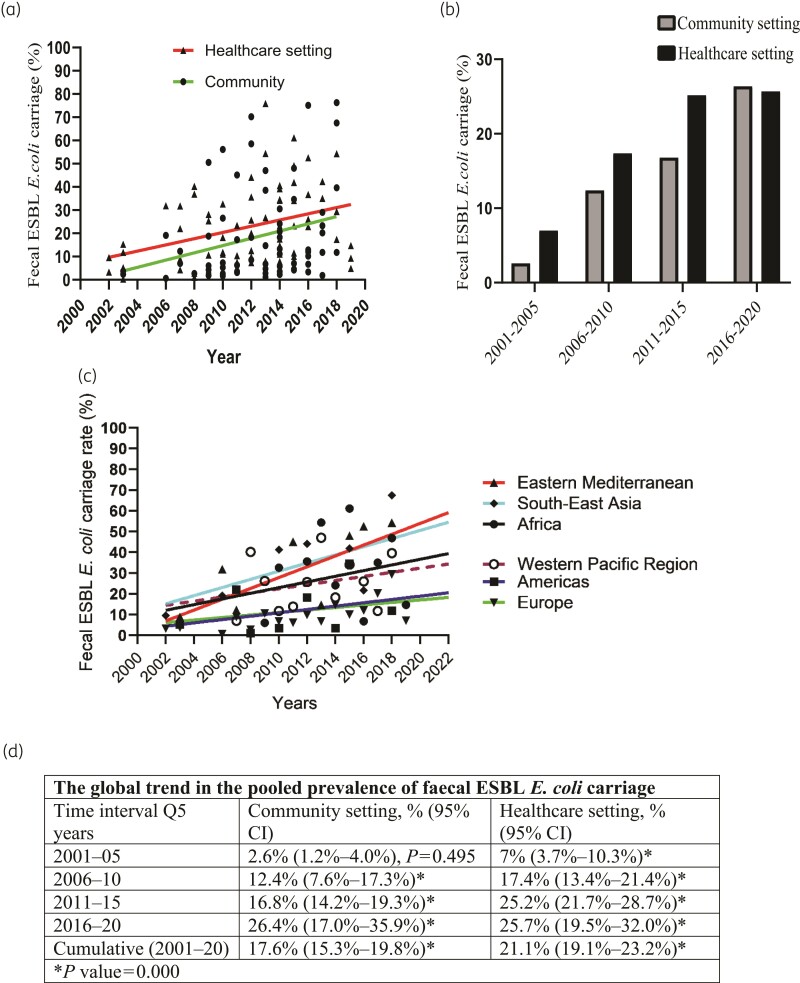
The global and regional trends in the prevalence of faecal ESBL *E. coli* carriage. (a) Linear regression plots showing the global trend in the carriage rate in the community (1.5% yearly increase, *P *= 0.027) and healthcare settings (1.3% annual rise, *P *= 0.003). (b) A bar graph depicting pooled prevalence by 5 year intervals of the study period (forest plots in Figures S4 and S5). In (c), studies of both community and healthcare settings were combined to show the regional trend in the six WHO regions (*P* values were not significant (>0.05) for all the regions). (d) A summary of global trend in human intestinal ESBL *E. coli* carriage with 95% CI as obtained from forest plots in Figures S4 and S5. *P* values are the *P* values for heterogeneity. Note: in (b) and (d), for the year interval 2001–05 there were only two studies for community setting and this might result in underestimation of the real prevalence.

Globally, and in each WHO region, the prevalence of human intestinal ESBL *E. coli* carriage showed a progressive increase from 2000 to 2021 (Figure 4a and c). Based on an estimation projection from linear regression analysis, and as shown in Figure 4(a), the global prevalence in the intestinal carriage of ESBL *E. coli* in the community was rising at a faster rate (a 1.5% yearly increase) than in the healthcare settings (1.3% annual rise). The global intestinal carriage rate of ESBL *E. coli* in the community increased 10-fold from 2.6% (95% CI, 1.2%–4.0%) in 2001–05 to 26.4% (95% CI, 17.0%–35.9%) in 2016–20 (Figure 4b and d, Figure S4), whereas in the healthcare setting, it increased from 7% (95% CI, 3.7%–10.3%) in 2001–05 to 25.7% (19.5%–32.0%) in 2016–20 (Figure 4b and d, Figure S5).

### Analysis of correlation between human intestinal ESBL E. coli carriage rates and duration of stay in healthcare setting

**Figure 5.**
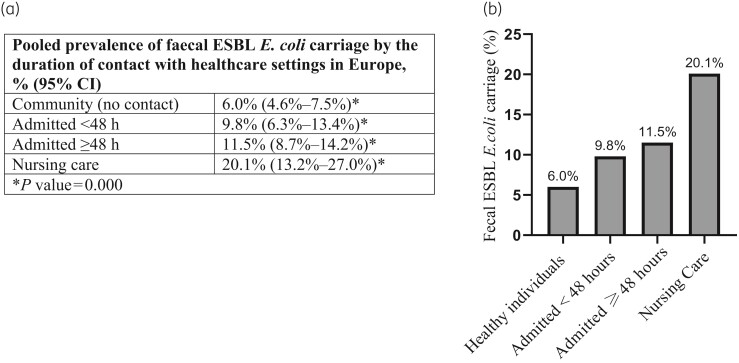
Comparison of faecal ESBL *E. coli* carriage rate between healthy individuals and inpatients in Europe. Carriage rates among inpatients increased with increasing duration of stay in healthcare settings. Summary table (a) and bar graph (b) were based on the meta-analysis forest plot in Figure S6. *P* values are the *P* values for heterogeneity. In this subgroup analysis, the absolute numbers of participants (ESBL *E. coli* positive/total screened) in Europe were: community setting (887/15168), admitted <48 h (1254/11983), admitted ≥48 h (1445/16589) and nursing care (671/3892).

An interesting finding, based on data from Europe, was that faecal ESBL *E. coli* colonization increased with increasing duration of contact/stay in a healthcare setting (Figure 5, Figure S6). Particularly, the prevalence of faecal ESBL *E. coli* colonization among patients admitted for more than 48 h was double the prevalence in healthy individuals, and in nursing care residents it was 3-fold higher than the prevalence in healthy individuals living in the community (Figure 5, Figure S6). Note, the other WHO regions had an insufficient number of studies for such analysis (Figure 2).

## Discussion

In this study, based on 73 318 samples, 21.1% of inpatients in healthcare settings and 17.6% of healthy individuals in the community worldwide carried MDR ESBL *E. coli* bacteria in their intestines. While Europe and the Americas had the lowest colonization rate, all the other WHO regions had a carriage rate of above 20% in both community and healthcare settings. Over the past 20 years (2000–21), the global human intestinal ESBL *E. coli* carriage rate increased steadily in both healthcare and community settings. The upward trend was observed in each of the six WHO regions. The rate of increase appeared to be higher in the community than in healthcare settings, and colonization rates in the community were approaching values in the healthcare areas. The reason for a slower pace of rise in healthcare settings is unknown, although we think this could be due to the practice of standard precautions (note, contact precautions had no added benefit over standard precautions^26,27^).

To our knowledge, this is the first study that has determined the global prevalence of the human intestinal carriage of ESBL *E. coli* in healthcare settings. We showed that at least one in five inpatients (21.1%) worldwide were carriers. The global carriage rate in the community (17.6%) was close to that reported in our previous publication (16.5%) (with the slight increase likely being the result of inclusion of more recent studies).^1^ By WHO region, South-East Asia had the highest carriage rate (35.1%) in the community, while the Eastern Mediterranean had the highest carriage rate (45.6%) in healthcare settings.

Although confidence intervals overlap, the global intestinal ESBL *E. coli* carriage rate appeared higher in healthcare than in community settings (Figures 4a and 5a). Regardless, there was a clear and statistically significant higher carriage rate in healthcare than community settings in Europe (where the number of included studies was large enough). However, in certain regions, such as the Americas and South-East Asia (regions with the lowest number of studies) and the Western Pacific, the carriage rate in the community appeared to be higher than in healthcare settings (Figure 3b and c). These findings may be attributable to a spatial ‘maldistribution’ of the individual studies used to calculate the pooled prevalence. For example, for the Americas, the regional pooled prevalence appeared higher in the community than healthcare setting. This was because, in this region, most of the individual studies describing prevalence in the community were from South America (area of high colonization), whereas most of the individual studies that measured carriage rate in healthcare settings were from North America (area of low colonization). We confirmed this with further sub-meta-analysis by sub-region of the Americas, which showed ESBL *E. coli* colonization rates actually appeared higher in healthcare than community settings in South America (Figure S7) and North America (Figure S8) (values were statistically insignificant). Sub-meta-analysis findings by sub-regions of Europe and the West Pacific are provided in the supplementary materials (Figures S9–S13) for further comparison.

This study also found that prolonged stay in a healthcare setting was associated with an increase in human intestinal colonization by ESBL *E. coli*. We demonstrated this using data from Europe, where nearly half of all the included studies in the healthcare setting were undertaken. This finding is consistent with other studies which found that prolonged hospitalization was an independent risk factor for intestinal ESBL *E. coli* colonization.^9,14^ Further, as antibiotics are commonly used in healthcare settings, the resulting gut dysbiosis (loss of gut colonization resistance) coupled with longer exposure to a high-prevalence healthcare setting (during a prolonged hospital stay) could provide a synergistic combination for increased acquisition.

This study has several limitations. First, as mentioned above, overestimation or underestimation of the global and regional pooled prevalence could result from the spatial ‘maldistribution’ of the individual studies included in the meta-analysis. For example, certain WHO regions might consist of countries with low and high colonization rates, and hence regional carriage rates in the community might appear higher than in healthcare settings. This occurs whenever a preponderance of studies included to calculate regional pooled prevalence for the community setting are from countries of high prevalence, while the studies used to measure regional carriage rates for the healthcare setting are from countries of low prevalence. A similar problem was seen when we grouped studies every 5 years to show the global trend. For instance, for the years 2016–20, the global cumulative prevalence in the community (26.4%) appeared higher than the values in healthcare settings (25.7%). However, our linear regression analysis found that the global human ESBL *E. coli* carriage in healthcare settings was always higher than in the community setting for all the years (2000–21) (Figure 4a). Hence, linear regression was used to offset such an intrinsic bias during the grouping of studies for pooled prevalence. In addition, there was a limited number of studies in some WHO regions.

Laboratory methods of ESBL identification improved over the years and this might have an influence on the rising trend in ESBL *E. coli* carriage. Besides, the techniques and sensitivity of the tests used might also differ in different regions of the world, leading to differences in ESBL *E. coli* prevalence. Publication bias (Figure S14), selection bias and the setting of studies (usually with interactions with the healthcare system) might have resulted in some overestimation of the prevalence in the community setting. For example, the total number of participants used to calculate the prevalence in the community included individuals who visited a health facility for a routine wellness check-up, but those individuals may have had prior interactions with a healthcare facility. The other limitation of this study is that the analysis was limited to the predominant ESBL-producing species—*E. coli*. The comparative prevalence of other Enterobacteriaceae needs to be addressed in future studies. Finally, the location of sample collection (community versus hospital versus nursing care) and the increased age of the nursing care residents may have introduced bias not accounted for in the calculation of the prevalence in the healthcare setting.^28^

### Conclusions

Global and regional human intestinal ESBL *E. coli* carriage is increasing in both community and healthcare settings. Carriage rates were generally higher in healthcare than in community settings. Based on data from Europe, where the most robust data were available, the faecal ESBL *E. coli* carriage rate among inpatients admitted for ≥48 h and nursing home residents was 2- and 3-fold, respectively, compared with the prevalence in healthy individuals living in the community. Key relevant health organizations should perform surveillance and implement preventive measures to address the spread of ESBL *E. coli* in both settings.

## Supplementary Material

dlac048_Supplementary_Data

## Data sharing

All data are available in the manuscript or the supplementary materials.

## Supplementary data

Tables S1–S3 and Figures S1–S14 are available as Supplementary data at *JAC-AMR* Online.

## References

[1] Bezabih YM , Sabiiti W, Alamneh E et al The global prevalence and trend of human intestinal carriage of ESBL-producing *Escherichia coli* in the community. J Antimicrob Chemother 2021; 76: 22–9. 33305801 10.1093/jac/dkaa399

[2] Melzer M , Petersen I. Mortality following bacteraemic infection caused by extended spectrum β-lactamase (ESBL) producing *E. coli* compared to non-ESBL producing *E. coli*. J Infect 2007; 55: 254–9. 17574678 10.1016/j.jinf.2007.04.007

[3] Sakran W , Smolkin V, Odetalla A et al Community-acquired urinary tract infection in hospitalized children: etiology and antimicrobial resistance. A comparison between first episode and recurrent infection. Clin Pediatr (Phila) 2015; 54: 479–83. 25385933 10.1177/0009922814555974

[4] Chervet D , Lortholary O, Zahar J-R et al Antimicrobial resistance in community-acquired urinary tract infections in Paris in 2015. Med Mal Infect 2018; 48: 188–92. 29054298 10.1016/j.medmal.2017.09.013

[5] Ranjan Dash N , Albataineh MT, Alhourani N et al Community-acquired urinary tract infections due to extended-spectrum β -lactamase-producing organisms in United Arab Emirates. Travel Med Infect Dis 2018; 22: 46–50. 29409967 10.1016/j.tmaid.2018.01.007

[6] Al-Mayahie S , Al Kuriashy JJ. Distribution of ESBLs among *Escherichia coli* isolates from outpatients with recurrent UTIs and their antimicrobial resistance. J Infect Dev Ctries 2016; 10: 575–83. 27367005 10.3855/jidc.6661

[7] Ouchar Mahamat O , Tidjani A, Lounnas M et al Fecal carriage of extended-spectrum β-lactamase-producing Enterobacteriaceae in hospital and community settings in Chad. Antimicrob Resist Infect Control 2019; 8: 169. 31695911 10.1186/s13756-019-0626-z PMC6824111

[8] Ko YJ , Moon H-W, Hur M et al Fecal carriage of extended-spectrum β-lactamase-producing Enterobacteriaceae in Korean community and hospital settings. Infection 2013; 41: 9–13. 22723075 10.1007/s15010-012-0272-3

[9] Kurz MSE , Bayingana C, Ndoli JM et al Intense pre-admission carriage and further acquisition of ESBL-producing Enterobacteriaceae among patients and their caregivers in a tertiary hospital in Rwanda. Trop Med Int Health 2017; 22: 210–20. 27935649 10.1111/tmi.12824

[10] van den Bunt G , van Pelt W, Hidalgo L et al Prevalence, risk factors and genetic characterisation of extended-spectrum β-lactamase and carbapenemase-producing Enterobacteriaceae (ESBL-E and CPE): a community-based cross-sectional study, the Netherlands, 2014 to 2016. Euro Surveill 2019; 24: 1800594. 10.2807/1560-7917.ES.2019.24.41.1800594 PMC6794991 31615600

[11] Arnan M , Gudiol C, Calatayud L et al Risk factors for, and clinical relevance of, faecal extended-spectrum β-lactamase producing *Escherichia coli* (ESBL-EC) carriage in neutropenic patients with haematological malignancies. Eur J Clin Microbiol Infect Dis 2011; 30: 355–60. 21052757 10.1007/s10096-010-1093-x

[12] Zhang H , Zhou Y, Guo S et al High prevalence and risk factors of fecal carriage of CTX-M type extended-spectrum β-lactamase-producing Enterobacteriaceae from healthy rural residents of Taian, China. Front Microbiol 2015; 6: 239. 25870591 10.3389/fmicb.2015.00239 PMC4376004

[13] Reuland EA , Al Naiemi N, Kaiser AM et al Prevalence and risk factors for carriage of ESBL-producing Enterobacteriaceae in Amsterdam. J Antimicrob Chemother 2016; 71: 1076–82. 26755493 10.1093/jac/dkv441 PMC4790620

[14] Kizilates F , Yakupogullari Y, Berk H et al Risk factors for fecal carriage of extended-spectrum β-lactamase-producing and carbapenem-resistant *Escherichia coli* and *Klebsiella pneumoniae* strains among patients at hospital admission. Am J Infect Control 2020; 49: 333–339. 32763346 10.1016/j.ajic.2020.07.035

[15] Karanika S , Karantanos T, Arvanitis M et al Fecal colonization with extended-spectrum β-lactamase–producing Enterobacteriaceae and risk factors among healthy individuals: a systematic review and metaanalysis. Clin Infect Dis 2016; 63: 310–8. 27143671 10.1093/cid/ciw283

[16] PRISMA . http://prisma-statement.org/prismastatement/Checklist.aspx.

[17] Jørgensen SB , Søraas A, Sundsfjord A et al Fecal carriage of extended spectrum β-lactamase producing *Escherichia coli* and *Klebsiella pneumoniae* after urinary tract infection – a three year prospective cohort study. PLoS One 2017; 12: e0173510. 28267783 10.1371/journal.pone.0173510 PMC5340397

[18] Rodríguez-Baño J , Alcalá JC, Cisneros JM et al Community infections caused by extended-spectrum β-lactamase–producing *Escherichia coli*. Arch Intern Med 2008; 168: 1897–902. 18809817 10.1001/archinte.168.17.1897

[19] National Heart, Lung, and Blood Institute (NHLBI) . Study Quality Assessment Tools. https://www.nhlbi.nih.gov/health-topics/study-quality-assessment-tools.

[20] DerSimonian R , Laird N. Meta-analysis in clinical trials. Control Clin Trials 1986; 7: 177–88. 3802833 10.1016/0197-2456(86)90046-2

[21] WHO Definition of Region Groupings . 2013. https://www.who.int/about/who-we-are/regional-offices.

[22] Fazel S , Khosla V, Doll H et al The prevalence of mental disorders among the homeless in Western countries: systematic review and meta-regression analysis. PLoS Med 2008; 5: e225. 19053169 10.1371/journal.pmed.0050225 PMC2592351

[23] Nyaga VN , Arbyn M, Aerts M. Metaprop: a Stata command to perform meta-analysis of binomial data. Arch Public Health 2014; 72: 39. 25810908 10.1186/2049-3258-72-39 PMC4373114

[24] Egger M , Davey Smith G, Schneider M et al Bias in meta-analysis detected by a simple, graphical test. BMJ 1997; 315: 629–34. 9310563 10.1136/bmj.315.7109.629 PMC2127453

[25] Wallace BC , Lajeunesse MJ, Dietz G et al Open MEE: intuitive, open-source software for meta-analysis in ecology and evolutionary biology. Methods Ecol Evol 2017; 8: 941–7.

[26] Tschudin-Sutter S , Lucet J-C, Mutters NT et al Contact precautions for preventing nosocomial transmission of extended-spectrum β lactamase–producing *Escherichia coli*: a point/counterpoint review. Clin Infect Dis 2017; 65: 342–7. 28379311 10.1093/cid/cix258

[27] Maechler F , Schwab F, Hansen S et al Contact isolation versus standard precautions to decrease acquisition of extended-spectrum β-lactamase-producing Enterobacterales in non-critical care wards: a cluster-randomised crossover trial. Lancet Infect Dis 2020; 20: 575–84. 32087113 10.1016/S1473-3099(19)30626-7

[28] Mshana SE , Falgenhauer L, Mirambo MM et al Predictors of *bla*_CTX-M-15_ in varieties of *Escherichia coli* genotypes from humans in community settings in Mwanza, Tanzania. BMC Infect Dis 2016; 16: 187. 27129719 10.1186/s12879-016-1527-x PMC4850702

[29] Medboua-Benbalagh C , Touati A, Kermas R et al Fecal carriage of extended-spectrum β-lactamase-producing Enterobacteriaceae strains is associated with worse outcome in patients hospitalized in the pediatric oncology unit of Beni-Messous Hospital in Algiers, Algeria. Microb Drug Resist 2017; 23: 757–63. 28095119 10.1089/mdr.2016.0153

[30] Ouédraogo A-S , Sanou S, Kissou A et al Fecal carriage of Enterobacteriaceae producing extended-spectrum β-lactamases in hospitalized patients and healthy community volunteers in Burkina Faso. Microb Drug Resist 2017; 23: 63–70. 27092971 10.1089/mdr.2015.0356

[31] Lonchel CM , Melin P, Gangoué-Piéboji J et al Extended-spectrum β-lactamase-producing Enterobacteriaceae in Cameroonian hospitals. Eur J Clin Microbiol Infect Dis 2013; 32: 79–87. 22886058 10.1007/s10096-012-1717-4

[32] Aklilu A , Manilal A, Ameya G et al Gastrointestinal tract colonization rate of extended-spectrum β-lactamase- and carbapenemase-producing Enterobacteriaceae and associated factors among hospitalized patients in Arba Minch General Hospital, Arba Minch, Ethiopia. Infect Drug Resist 2020; 13: 1517–26. 32547121 10.2147/IDR.S239092 PMC7250175

[33] Desta K , Woldeamanuel Y, Azazh A et al High gastrointestinal colonization rate with extended-spectrum β-lactamase-producing Enterobacteriaceae in hospitalized patients: emergence of carbapenemase-producing *K. pneumoniae* in Ethiopia. PLoS One 2016; 11: e0161685. 27574974 10.1371/journal.pone.0161685 PMC5004900

[34] Falgenhauer L , Imirzalioglu C, Oppong K et al Detection and characterization of ESBL-producing *Escherichia coli* from humans and poultry in Ghana. Front Microbiol 2019; 9: 3358. 30697208 10.3389/fmicb.2018.03358 PMC6340976

[35] Isendahl J , Turlej-Rogacka A, Manjuba C et al Fecal carriage of ESBL-producing *E. coli* and *K. pneumoniae* in children in Guinea-Bissau: a hospital-based cross-sectional study. PLoS One 2012; 7: e51981. 23284838 10.1371/journal.pone.0051981 PMC3527401

[36] Rakotomalala R , Rahariniainasoa A, Rakotonindrina F et al Intestinal carriage of ESBL-E among sick children in Mahajanga, Madagascar. BMR Microbiol 2019: 1–3.

[37] Sallem R B , Slama K B, Estepa V et al Detection of CTX-M-15-producing *Escherichia coli* isolates of lineages ST410-A, ST617-A and ST354-D in faecal samples of hospitalized patients in a Mauritanian hospital. J Chemother 2015; 27: 114–6. 24548094 10.1179/1973947814Y.0000000172

[38] Founou RC , Founou LL, Essack SY. Extended spectrum β-lactamase mediated resistance in carriage and clinical gram-negative ESKAPE bacteria: a comparative study between a district and tertiary hospital in South Africa. Antimicrob Resist Infect Control 2018; 7: 134. 30473784 10.1186/s13756-018-0423-0 PMC6237030

[39] Akinduti PA , Olasehinde GI, Oluwaseun E et al Fecal carriage and phylodiversity of community-acquired *bla*_TEM_ enteric bacilli in Southwest Nigeria. Infect Drug Resist 2018; 11: 2425–33. 30568469 10.2147/IDR.S178243 PMC6267359

[40] Herindrainy P , Rabenandrasana MAN, Andrianirina ZZ et al Acquisition of extended spectrum β-lactamase-producing Enterobacteriaceae in neonates: a community based cohort in Madagascar. PLoS One 2018; 13: e0193325. 29494706 10.1371/journal.pone.0193325 PMC5832238

[41] Büdel T , Kuenzli E, Clément M et al Polyclonal gut colonization with extended-spectrum cephalosporin- and/or colistin-resistant Enterobacteriaceae: a normal status for hotel employees on the island of Zanzibar, Tanzania. J Antimicrob Chemother 2019; 74: 2880–90. 31361004 10.1093/jac/dkz296

[42] Cortés-Cortés G , Lozano-Zarain P, Torres C et al Extended-spectrum β-lactamase-producing *Escherichia coli* isolated from healthy humans in Mexico, including subclone ST131-B2-O25:H4-H30-Rx. J Glob Antimicrob Resist 2017; 9: 130–4. 28552831 10.1016/j.jgar.2017.02.014

[43] Salinas L , Loayza F, Cárdenas P et al Environmental spread of extended spectrum β-lactamase (ESBL) producing *Escherichia coli* and ESBL genes among children and domestic animals in Ecuador. Environ Health Perspect 2021; 129: 027007. 10.1289/EHP7729 PMC7899495 33617318

[44] Woerther P-L , Angebault C, Jacquier H et al Characterization of fecal extended-spectrum-β-lactamase-producing *Escherichia coli* in a remote community during a long time period. Antimicrob Agents Chemother 2013; 57: 5060–6. 23917313 10.1128/AAC.00848-13 PMC3811437

[45] Islam S , Selvarangan R, Kanwar N et al Intestinal carriage of third-generation cephalosporin-resistant and extended-spectrum β-lactamase-producing Enterobacteriaceae in healthy US children. J Ped Infect Dis Soc 2018; 7: 234–40. 10.1093/jpids/pix045 PMC5820225 28992133

[46] Weisenberg SA , Mediavilla JR, Chen L et al Extended spectrum β-lactamase-producing Enterobacteriaceae in international travelers and non-travelers in New York City. PLoS One 2012; 7: e45141. 23028808 10.1371/journal.pone.0045141 PMC3447858

[47] Araque M , Labrador I. Prevalence of fecal carriage of CTX-M-15 β-lactamase-producing *Escherichia coli* in healthy children from a rural Andean village in Venezuela. Osong Public Health Res Perspect 2018; 9: 9–15. 29503800 10.24171/j.phrp.2018.9.1.03 PMC5831685

[48] Vasques MRG , Bello AR, Lamas CC et al β-Lactamase producing enterobacteria isolated from surveillance swabs of patients in a cardiac intensive care unit in Rio de Janeiro, Brazil. Braz J Infect Dis 2011; 15: 28–33. 21412586

[49] Harris AD , Kotetishvili M, Shurland S et al How important is patient-to-patient transmission in extended-spectrum β-lactamase *Escherichia coli* acquisition. Am J Infect Control 2007; 35: 97–101. 17327188 10.1016/j.ajic.2006.09.011

[50] Han JH , Nachamkin I, Zaoutis TE et al Risk factors for gastrointestinal tract colonization with extended-spectrum β-lactamase (ESBL)–producing *Escherichia coli* and *Klebsiella* species in hospitalized patients. Infect Control Hosp Epidemiol 2012; 33: 1242–5. 23143363 10.1086/668443 PMC3983276

[51] Hashemizadeh Z , Kalantar-Neyestanaki D, Mansouri S. Clonal relationships, antimicrobial susceptibilities, and molecular characterization of extended-spectrum β-lactamase-producing *Escherichia coli* isolates from urinary tract infections and fecal samples in Southeast Iran. Rev Soc Bras Med Trop 2018; 51: 44–51. 29513841 10.1590/0037-8682-0080-2017

[52] Abdul Rahman EM , El-Sherif RH. High rates of intestinal colonization with extended-spectrum lactamase-producing Enterobacteriaceae among healthy individuals. J Investig Med 2011; 59: 1284–6. 10.2130/JIM.0b013e318238748e 22068018

[53] Fam NS , Defasque S, Bert F et al Faecal carriage of extended-spectrum β-lactamase (ESBL)-producing enterobacteria in liver disease patients from two hospitals in Egypt and France: a comparative epidemiological study. Epidemiol Infect 2015; 143: 1247–55. 25036046 10.1017/S0950268814001812 PMC9507165

[54] Aghamohammad S , Badmasti F, Shirazi AS et al Considerable rate of putative virulent phylo-groups in fecal carriage of extended-spectrum β-lactamase producing *Escherichia coli*. Infect Genet Evol 2019; 73: 184–9. 31054921 10.1016/j.meegid.2019.04.035

[55] Hashemizadeh Z , Mohebi S, Kalantar-Neyestanaki D et al Prevalence of plasmid-mediated quinolone resistance and ESBLs genes in *Escherichia coli* isolated from urinary tract infections and fecal samples in Southeast Iran. Gene Reports 2019; 17: 100487.

[56] Moubareck C , Daoud Z, Hakime NI et al Countrywide spread of community- and hospital-acquired extended-spectrum -lactamase (CTX-M-15)-producing Enterobacteriaceae in Lebanon. J Clin Microbiol 2005; 43: 3309–13. 16000453 10.1128/JCM.43.7.3309-3313.2005 PMC1169093

[57] Hijazi SM , Fawzi MA, Ali FM et al Multidrug-resistant ESBL-producing Enterobacteriaceae and associated risk factors in community infants in Lebanon. J Infect Dev Ctries 2016; 10: 947–55. 27694727 10.3855/jidc.7593

[58] Daoud Z , Moubareck C, Hakime N et al Extended spectrum β-lactamase producing Enterobacteriaceae in Lebanese ICU patients: epidemiology and patterns of resistance. J Gen Appl Microbiol 2006; 52: 169–78. 16960333 10.2323/jgam.52.169

[59] Barguigua A , Ouair H, El Otmani F et al Fecal carriage of extended-spectrum β-lactamase-producing Enterobacteriaceae in community setting in Casablanca. Infect Dis (Lond) 2015; 47: 27–32. 25329550 10.3109/00365548.2014.961542

[60] Kader AA , Kamath KA. Faecal carriage of extended-spectrum β-lactamase-producing bacteria in the community. East Mediterr Health J 2009; 15: 1365–70. 20218126

[61] Elkersh T , Marie MA, Al-Sheikh YA et al Prevalence of fecal carriage of extended-spectrum- and metallo-β-lactamase-producing Gram-negative bacteria among neonates born in a hospital setting in central Saudi Arabia. Ann Saudi Med 2015; 35: 240–7. 26409799 10.5144/0256-4947.2015.240 PMC6074465

[62] Kader AA , Kumar A, Kamath KA. Fecal carriage of extended-spectrum β-lactamase-producing *Escherichia coli* and *Klebsiella pneumoniae* in patients and asymptomatic healthy individuals. Infect Control Hosp Epidemiol 2007; 28: 1114–6. 17932839 10.1086/519865

[63] Ferjani S , Saidani M, Hamzaoui Z et al Community fecal carriage of broad-spectrum cephalosporin-resistant *Escherichia coli* in Tunisian children. Diagn Microbiol Infect Dis 2017; 87: 188–92. 27856044 10.1016/j.diagmicrobio.2016.03.008

[64] Valenza G , Nickel S, Pfeifer Y et al Extended-spectrum-β-lactamase-producing *Escherichia coli* as intestinal colonizers in the German community. Antimicrob Agents Chemother 2014; 58: 1228–30. 24295972 10.1128/AAC.01993-13 PMC3910888

[65] Lübbert C , Straube L, Stein C et al Colonization with extended-spectrum β-lactamase-producing and carbapenemase-producing Enterobacteriaceae in international travelers returning to Germany. Int J Med Microbiol 2015; 305: 148–56. 25547265 10.1016/j.ijmm.2014.12.001

[66] Guimarães B , Barreto Â, Radhouani H et al Genetic detection of extended-spectrum β-lactamase–containing *Escherichia coli* isolates and vancomycin-resistant enterococci in fecal samples of healthy children. Microb Drug Resist 2009; 15: 211–6. 19728780 10.1089/mdr.2009.0910

[67] Latour K , Huang T-D, Jans B et al Prevalence of multidrug-resistant organisms in nursing homes in Belgium in 2015. PLoS One 2019; 14: e0214327. 30921364 10.1371/journal.pone.0214327 PMC6438666

[68] Ruh E , Zakka J, Hoti K et al Extended-spectrum β-lactamase, plasmid-mediated AmpC β-lactamase, fluoroquinolone resistance, and decreased susceptibility to carbapenems in Enterobacteriaceae: fecal carriage rates and associated risk factors in the community of Northern Cyprus. Antimicrob Resist Infect Control 2019; 8: 98. 31198531 10.1186/s13756-019-0548-9 PMC6558775

[69] Dall LB , Lausch KR, Gedebjerg A et al Do probiotics prevent colonization with multi-resistant Enterobacteriaceae during travel? A randomized controlled trial. Travel Med Infect Dis 2019; 27: 81–6. 30508633 10.1016/j.tmaid.2018.11.013

[70] Bert F , Larroque B, Paugam-Burtz C et al Pretransplant fecal carriage of extended-spectrum β-lactamase-producing Enterobacteriaceae and infection after liver transplant, France. Emerging Infect Dis 2012; 18: 908–16. 10.3201/eid1806.110139 PMC3358139 22607885

[71] Leflon-Guibout V , Blanco J, Amaqdouf K et al Absence of CTX-M enzymes but high prevalence of clones, including clone ST131, among fecal *Escherichia coli* isolates from healthy subjects living in the area of Paris, France. J Clin Microbiol 2008; 46: 3900–5. 18842941 10.1128/JCM.00734-08 PMC2593250

[72] Janvier F , Mérens A, Delaune D et al Portage digestif d’entérobactéries résistantes aux céphalosporines de troisième génération dans une population d’adultes jeunes asymptomatiques : évolution entre 1999 et 2009. Pathol Biol (Paris) 2011; 59: 97–101. 20828938 10.1016/j.patbio.2010.07.012

[73] Pilmis B , Cattoir V, Lecointe D et al Carriage of ESBL-producing Enterobacteriaceae in French hospitals: the PORTABLSE study. J Hosp Infect 2018; 98: 247–52. 29222035 10.1016/j.jhin.2017.11.022

[74] Vidal-Navarro L , Pfeiffer C, Bouziges N et al Faecal carriage of multidrug-resistant Gram-negative bacilli during a non-outbreak situation in a French university hospital. J Antimicrob Chemother 2010; 65: 2455–8. 20813808 10.1093/jac/dkq333

[75] Boutet-Dubois A , Pantel A, Prère M-F et al Faecal carriage of oxyiminocephalosporin-resistant Enterobacteriaceae among paediatric units in different hospitals in the south of France. Eur J Clin Microbiol Infect Dis 2013; 32: 1063–8. 23494770 10.1007/s10096-013-1851-7

[76] Le Bastard Q , Chapelet G, Birgand G et al Gut microbiome signatures of nursing home residents carrying Enterobacteria producing extended-spectrum β-lactamases. Antimicrob Resist Infect Control 2020; 9: 107. 32665016 10.1186/s13756-020-00773-y PMC7359458

[77] Jolivet S , Vaillant L, Poncin T et al Prevalence of carriage of extended-spectrum β-lactamase-producing enterobacteria and associated factors in a French hospital. Clin Microbiol Infect 2018; 24: 1311–4. 29549056 10.1016/j.cmi.2018.03.008

[78] Meyer E , Gastmeier P, Kola A et al Pet animals and foreign travel are risk factors for colonisation with extended-spectrum β-lactamase-producing *Escherichia coli*. Infection 2012; 40: 685–7. 22971936 10.1007/s15010-012-0324-8

[79] Vehreschild MJGT , Hamprecht A, Peterson L et al A multicentre cohort study on colonization and infection with ESBL-producing Enterobacteriaceae in high-risk patients with haematological malignancies. J Antimicrob Chemother 2014; 69: 3387–92. 25103492 10.1093/jac/dku305

[80] Hamprecht A , Rohde AM, Behnke M et al Colonization with third-generation cephalosporin-resistant Enterobacteriaceae on hospital admission: prevalence and risk factors. J Antimicrob Chemother 2016; 71: 2957–63. 27317445 10.1093/jac/dkw216

[81] Reinheimer C , Keppler OT, Stephan C et al Elevated prevalence of multidrug-resistant gram-negative organisms in HIV positive men. BMC Infect Dis 2017; 17: 206. 28288577 10.1186/s12879-017-2286-z PMC5347171

[82] Arvand M , Ruscher C, Bettge-Weller G et al Prevalence and risk factors for colonization by Clostridium difficile and extended-spectrum β-lactamase-producing Enterobacteriaceae in rehabilitation clinics in Germany. J Hosp Infect 2018; 98: 14–20. 28705583 10.1016/j.jhin.2017.07.004

[83] Ebrahimi F , Mózes J, Mészáros J et al Asymptomatic faecal carriage of ESBL producing enterobacteriaceae in Hungarian healthy individuals and in long-term care applicants: a shift towards CTX-M producers in the community. Infect Dis (Lond) 2016; 48: 557–9. 26982242 10.3109/23744235.2016.1155734

[84] Ebrahimi F , Mózes J, Monostori J et al Comparison of rates of fecal colonization with extended-spectrum β-lactamase-producing enterobacteria among patients in different wards, outpatients and medical students. Microbiol Immunol 2016; 60: 285–94. 26959958 10.1111/1348-0421.12373

[85] Adler A , Gniadkowski M, Baraniak A et al Transmission dynamics of ESBL-producing *Escherichia coli* clones in rehabilitation wards at a tertiary care centre. Clin Microbiol Infect 2012; 18: E497–505. 22963432 10.1111/j.1469-0691.2012.03999.x

[86] Giufrè M , Ricchizzi E, Accogli M et al Colonization by multidrug-resistant organisms in long-term care facilities in Italy: a point-prevalence study. Clin Microbiol Infect 2017; 23: 961–7. 28412380 10.1016/j.cmi.2017.04.006

[87] March A , Aschbacher R, Dhanji H et al Colonization of residents and staff of a long-term-care facility and adjacent acute-care hospital geriatric unit by multiresistant bacteria. Clin Microbiol Infect 2010; 16: 934–44. 19686277 10.1111/j.1469-0691.2009.03024.x

[88] Meletiadis J , Turlej-Rogacka A, Lerner A et al Amplification of antimicrobial resistance in gut flora of patients treated with ceftriaxone. Antimicrob Agents Chemother 2017; 61: e00473-17. 28807914 10.1128/AAC.00473-17 PMC5655041

[89] Willemsen I , Nelson J, Hendriks Y et al Extensive dissemination of extended spectrum β-lactamase-producing Enterobacteriaceae in a Dutch nursing home. Infect Control Hosp Epidemiol 2015; 36: 394–400. 25782893 10.1017/ice.2014.76

[90] Overdevest I , Haverkate M, Veenemans J et al Prolonged colonisation with *Escherichia coli* O25:ST131 versus other extended-spectrum β-lactamase-producing *E. coli* in a long-term care facility with high endemic level of rectal colonisation, the Netherlands, 2013 to 2014. Euro Surveill 2016; 21: 30376. 10.2807/1560-7917.ES.2016.21.42.30376 PMC5291152 27784530

[91] Kluytmans-van den Bergh MFQ , van Mens SP, Haverkate MR et al Quantifying hospital-acquired carriage of extended-spectrum β-lactamase-producing Enterobacteriaceae among patients in Dutch hospitals. Infect Control Hosp Epidemiol 2018; 39: 32–9. 29215330 10.1017/ice.2017.241

[92] Ulstad CR , Solheim M, Berg S et al Carriage of ESBL/AmpC-producing or ciprofloxacin non-susceptible *Escherichia coli* and *Klebsiella* spp. in healthy people in Norway. Antimicrob Resist Infect Control 2016; 5: 57. 28018582 10.1186/s13756-016-0156-x PMC5159956

[93] Sadowska-Klasa A , Piekarska A, Prejzner W et al Colonization with multidrug-resistant bacteria increases the risk of complications and a fatal outcome after allogeneic hematopoietic cell transplantation. Ann Hematol 2018; 97: 509–17. 29255911 10.1007/s00277-017-3205-5 PMC5797223

[94] Aires-de-Sousa M , Lopes E, Gonçalves ML et al Intestinal carriage of extended-spectrum β-lactamase-producing Enterobacteriaceae at admission in a Portuguese hospital. Eur J Clin Microbiol Infect Dis 2020; 39: 783–90. 31873863 10.1007/s10096-019-03798-3

[95] Valverde A , Coque TM, Sanchez-Moreno MP et al Dramatic increase in prevalence of fecal carriage of extended-spectrum β-lactamase-producing Enterobacteriaceae during nonoutbreak situations in Spain. J Clin Microbiol 2004; 42: 4769–75. 15472339 10.1128/JCM.42.10.4769-4775.2004 PMC522353

[96] Jiménez-Rámila C , López-Cerero L, Aguilar Martín MV et al Vagino-rectal colonization and maternal–neonatal transmission of Enterobacteriaceae producing extended-spectrum β-lactamases or carbapenemases: a cross-sectional study. J Hosp Infect 2019; 101: 167–74. 30248371 10.1016/j.jhin.2018.09.010

[97] Paniagua R , Valverde A, Coque TM et al Assessment of prevalence and changing epidemiology of extended-spectrum β-lactamase-producing Enterobacteriaceae fecal carriers using a chromogenic medium. Diagn Microbiol Infect Dis 2010; 67: 376–9. 20638607 10.1016/j.diagmicrobio.2010.03.012

[98] Calatayud L , Arnan M, Liñares J et al Prospective study of fecal colonization by extended-spectrum-β-lactamase-producing *Escherichia coli* in neutropenic patients with cancer. Antimicrob Agents Chemother 2008; 52: 4187–90. 18809942 10.1128/AAC.00367-08 PMC2573121

[99] Miró E , Mirelis B, Navarro F et al Surveillance of extended-spectrum β-lactamases from clinical samples and faecal carriers in Barcelona, Spain. J Antimicrob Chemother 2005; 56: 1152–5. 16244084 10.1093/jac/dki395

[100] Colmenarejo C , Hernández-García M, Muñoz-Rodríguez JR et al Prevalence and risks factors associated with ESBL-producing faecal carriage in a single long-term-care facility in Spain: emergence of CTX-M-24- and CTX-M-27-producing *Escherichia coli* ST131-H30R. J Antimicrob Chemother 2020; 75: 2480–4. 32542354 10.1093/jac/dkaa219

[101] Kaarme J , Molin Y, Olsen B et al Prevalence of extended-spectrum β-lactamase-producing Enterobacteriaceae in healthy Swedish preschool children. Acta Paediatr 2013; 102: 655–60. 23419070 10.1111/apa.12206

[102] Ny S , Löfmark S, Börjesson S et al Community carriage of ESBL-producing *Escherichia coli* is associated with strains of low pathogenicity: a Swedish nationwide study. J Antimicrob Chemother 2017; 72: 582–8. 27798205 10.1093/jac/dkw419

[103] Strömdahl H , Tham J, Melander E et al Prevalence of faecal ESBL carriage in the community and in a hospital setting in a county of Southern Sweden. Eur J Clin Microbiol Infect Dis 2011; 30: 1159–62. 21399889 10.1007/s10096-011-1202-5

[104] Chabok A , Tärnberg M, Smedh K et al Prevalence of fecal carriage of antibiotic-resistant bacteria in patients with acute surgical abdominal infections. Scand J Gastroenterol 2010; 45: 1203–10. 20521871 10.3109/00365521.2010.495417

[105] Blom A , Ahl J, Månsson F et al The prevalence of ESBL-producing Enterobacteriaceae in a nursing home setting compared with elderly living at home: a cross-sectional comparison. BMC Infect Dis 2016; 16: 111. 26944857 10.1186/s12879-016-1430-5 PMC4778276

[106] Andersson H , Lindholm C, Iversen A et al Prevalence of antibiotic-resistant bacteria in residents of nursing homes in a Swedish municipality: healthcare staff knowledge of and adherence to principles of basic infection prevention. Scand J Infect Dis 2012; 44: 641–9. 22680834 10.3109/00365548.2012.671956

[107] Kuenzli E , Jaeger VK, Frei R et al High colonization rates of extended-spectrum β-lactamase (ESBL)-producing *Escherichia coli* in Swiss travellers to South Asia– a prospective observational multicentre cohort study looking at epidemiology, microbiology and risk factors. BMC Infect Dis 2014; 14: 528. 25270732 10.1186/1471-2334-14-528 PMC4262238

[108] Tukenmez Tigen E , Tandogdu Z, Ergonul O et al Outcomes of fecal carriage of extended-spectrum β-lactamase after transrectal ultrasound–guided biopsy of the prostate. Urology 2014; 84: 1008–15. 25239255 10.1016/j.urology.2014.04.060

[109] Blane B , Brodrick HJ, Gouliouris T et al Comparison of 2 chromogenic media for the detection of extended-spectrum β-lactamase producing Enterobacteriaceae stool carriage in nursing home residents. Diagn Microbiol Infect Dis 2016; 84: 181–3. 26712266 10.1016/j.diagmicrobio.2015.11.008 PMC4769092

[110] Munday CJ , Whitehead GM, Todd NJ et al Predominance and genetic diversity of community- and hospital-acquired CTX-M extended-spectrum β-lactamases in York, UK. J Antimicrob Chemother 2004; 54: 628–33. 15294889 10.1093/jac/dkh397

[111] Mathai D , Kumar VA, Paul B et al Fecal carriage rates of extended-spectrum β-lactamase-producing *Escherichia coli* among antibiotic naive healthy human volunteers. Microb Drug Resist 2015; 21: 59–64. 25127253 10.1089/mdr.2014.0031

[112] Rousham EK , Unicomb L, Islam MA. Human, animal and environmental contributors to antibiotic resistance in low-resource settings: integrating behavioural, epidemiological and One Health approaches. Proc R Soc B 2018; 285:20180332. 10.1098/rspb.2018.0332 PMC5904322 29643217

[113] Babu R , Kumar A, Karim S et al Faecal carriage rate of extended-spectrum β-lactamase-producing Enterobacteriaceae in hospitalised patients and healthy asymptomatic individuals coming for health check-up. J Glob Antimicrob Resist 2016; 6: 150–3. 27530858 10.1016/j.jgar.2016.05.007

[114] Maharjan A , Bhetwal A, Shakya S et al Ugly bugs in healthy guts! Carriage of multidrug-resistant and ESBL-producing commensal Enterobacteriaceae in the intestine of healthy Nepalese adults. Infect Drug Resist 2018; 11: 547–54. 29731643 10.2147/IDR.S156593 PMC5927189

[115] Luvsansharav U-O , Hirai I, Nakata A et al Prevalence of and risk factors associated with faecal carriage of CTX-M -lactamase-producing Enterobacteriaceae in rural Thai communities. J Antimicrob Chemother 2012; 67: 1769–74. 22514260 10.1093/jac/dks118

[116] Mulki SS , Ramamurthy K, Bhat S. Fecal carriage of extended-spectrum β-lactamase-producing Enterobacteriaceae in intensive care unit patients. Indian J Crit Care Med 2017; 21: 525–7. 28904483 10.4103/ijccm.IJCCM_112_17 PMC5588488

[117] Severin JA , Lestari ES, Kloezen W et al Faecal carriage of extended-spectrum β-lactamase-producing Enterobacteriaceae among humans in Java, Indonesia, in 2001-2002. Trop Med Int Health 2012; 17: 455–61. 22248076 10.1111/j.1365-3156.2011.02949.x

[118] Kiddee A , Assawatheptawee K, Na-Udom A et al Risk factors for extended-spectrum β-lactamase-producing Enterobacteriaceae carriage in patients admitted to intensive care unit in a tertiary care hospital in Thailand. Microb Drug Resist 2019; 25: 1182–90. 31140920 10.1089/mdr.2018.0318

[119] Tian SF , Chen BY, Chu YZ et al Prevalence of rectal carriage of extended-spectrum β-lactamase-producing *Escherichia coli* among elderly people in community settings in China. Can J Microbiol 2008; 54: 781–5. 18772941 10.1139/w08-059

[120] Sadahira T , Wada K, Araki M et al Impact of selective media for detecting fluoroquinolone-insusceptible/extended-spectrum β-lactamase-producing *Escherichia coli* before transrectal prostate biopsy. Int J Urol 2017; 24: 842–7. 28929546 10.1111/iju.13447

[121] Nakane K , Kawamura K, Goto K et al Long-term colonization by *bla*_CTX-M_ -harboring *Escherichia coli* in healthy Japanese people engaged in food handling. Appl Environ Microbiol 2016; 82: 1818–27. 26746714 10.1128/AEM.02929-15 PMC4784029

[122] Nakayama T , Ueda S, Huong BTM et al Wide dissemination of extended-spectrum &beta;-lactamase-producing *Escherichia coli* in community residents in the Indochinese peninsula. Infect Drug Resist 2015; 8: 1–5. 25670909 10.2147/IDR.S74934 PMC4315533

[123] Kennedy K , Collignon P. Colonisation with *Escherichia coli* resistant to ‘critically important’ antibiotics: a high risk for international travellers. Eur J Clin Microbiol Infect Dis 2010; 29: 1501–6. 20835879 10.1007/s10096-010-1031-y

[124] Stuart RL , Kotsanas D, Webb B et al Prevalence of antimicrobial-resistant organisms in residential aged care facilities. Med J Aust 2011; 195: 530–3. 22060088 10.5694/mja11.10724

[125] Lim CJ , Cheng AC, Kennon J et al Prevalence of multidrug-resistant organisms and risk factors for carriage in long-term care facilities: a nested case–control study. J Antimicrob Chemother 2014; 69: 1972–80. 24710025 10.1093/jac/dku077

[126] Atterby C , Osbjer K, Tepper V et al Carriage of carbapenemase- and extended-spectrum cephalosporinase-producing *Escherichia coli* and *Klebsiella pneumoniae* in humans and livestock in rural Cambodia; gender and age differences and detection of *bla*_OXA-48_ in humans. Zoonoses Public Health 2019; 66: 603–17. 31264805 10.1111/zph.12612 PMC6852310

[127] Zhou Y , Wu X, Zhang J et al High prevalence of CTX-M β-lactamases in Enterobacteriaceae from healthy individuals in Guangzhou, China. Microb Drug Resist 2015; 21: 398–403. 25756950 10.1089/mdr.2014.0201

[128] Qin X , Hu F, Wu S et al Comparison of adhesin genes and antimicrobial susceptibilities between uropathogenic and intestinal commensal *Escherichia coli* strains. PLoS One 2013; 8: e61169. 23593422 10.1371/journal.pone.0061169 PMC3621879

[129] Li B , Sun J-Y, Liu Q-Z et al High prevalence of CTX-M β-lactamases in faecal *Escherichia coli* strains from healthy humans in Fuzhou, China. Scand J Infect Dis 2011; 43: 170–4. 21128708 10.3109/00365548.2010.538856

[130] Ni Q , Tian Y, Zhang L et al Prevalence and quinolone resistance of fecal carriage of extended-spectrum β-lactamase-producing *Escherichia coli* in 6 communities and 2 physical examination center populations in Shanghai, China. Diagn Microbiol Infect Dis 2016; 86: 428–33. 27681363 10.1016/j.diagmicrobio.2016.07.010

[131] Xu M , Fan Y, Wang M et al Characteristics of extended-spectrum β-lactamases-producing *Escherichia coli* in fecal samples of inpatients of Beijing Tongren Hospital. Jpn J Infect Dis 2017; 70: 290–4. 27795466 10.7883/yoken.JJID.2016.023

[132] Wen Z , Wei X, Xiao Y et al Intervention study of the association of antibiotic utilization measures with control of extended-spectrum β-lactamase (ESBL)-producing bacteria. Microbes Infect 2010; 12: 710–5. 20457272 10.1016/j.micinf.2010.04.015

[133] Kamei J , Yagihara Y, Kume H et al Prevalence and characteristics of fecal antimicrobial-resistant *Escherichia coli* in a cohort of Japanese men undergoing prostate biopsy. Int J Urol 2017; 24: 295–300. 28222483 10.1111/iju.13308

[134] Higa S , Sarassari R, Hamamoto K et al Characterization of CTX-M type ESBL-producing Enterobacteriaceae isolated from asymptomatic healthy individuals who live in a community of the Okinawa prefecture, Japan. J Infect Chemother 2019; 25: 314–7. 30292768 10.1016/j.jiac.2018.09.005

[135] Nakamura A , Komatsu M, Noguchi N et al Analysis of molecular epidemiologic characteristics of extended-spectrum β-lactamase (ESBL)-producing *Escherichia coli* colonizing feces in hospital patients and community dwellers in a Japanese city. J Infect Chemother 2016; 22: 102–7. 26705747 10.1016/j.jiac.2015.11.001

[136] Luvsansharav U-O , Hirai I, Niki M et al Fecal carriage of CTX-M β-lactamase-producing Enterobacteriaceae in nursing homes in the Kinki region of Japan. Infect Drug Resist 2013; 6: 67–70. 23900409 10.2147/IDR.S43868 PMC3724607

[137] Takano C , Seki M, Shiihara H et al Frequent isolation of extended-spectrum β-lactamase-producing bacteria from fecal samples of individuals with severe motor and intellectual disabilities. J Infect Chemother 2018; 24: 182–7. 29398476 10.1016/j.jiac.2017.10.003

[138] Kawamura K , Hayashi K, Matsuo N et al Prevalence of CTX-M-type extended-spectrum β-lactamase-producing *Escherichia coli* B2-O25-ST131 H30R among residents in nonacute care facilities in Japan. Microb Drug Resist 2018; 24: 1513–20. 29791251 10.1089/mdr.2018.0068

[139] Baljin B , Baldan G, Chimeddorj B et al Faecal carriage of Gram-negative multidrug-resistant bacteria among patients hospitalized in two centres in Ulaanbaatar, Mongolia. PLoS One 2016; 11: e0168146. 27942042 10.1371/journal.pone.0168146 PMC5152906

[140] Mo Y , Seah I, Lye PSP et al Relating knowledge, attitude and practice of antibiotic use to extended-spectrum β-lactamase-producing Enterobacteriaceae carriage: results of a cross-sectional community survey. BMJ Open 2019; 9: e023859. 10.1136/bmjopen-2018-023859 PMC6429736 30842108

[141] Joo E-J , Kim SJ, Baek M et al Fecal carriage of antimicrobial-resistant Enterobacteriaceae in healthy Korean adults. J Microbiol Biotechnol 2018; 28: 1178–84. 29913545 10.4014/jmb.1801.12060

[142] Wu P-C , Wang J-L, Hsueh P-R et al Prevalence and risk factors for colonization by extended-spectrum β-lactamase-producing or ST 131 *Escherichia coli* among asymptomatic adults in community settings in Southern Taiwan. Infect Drug Resist 2019; 12: 1063–71. 31118712 10.2147/IDR.S201086 PMC6506006

[143] Huang Y-S , Lai L-C, Chen Y-A et al Colonization with multidrug-resistant organisms among healthy adults in the community setting: prevalence, risk factors, and composition of gut microbiome. Front Microbiol 2020; 11: 1402. 32670243 10.3389/fmicb.2020.01402 PMC7328365

[144] Thi Quynh Nhi L , Thanh Tuyen H, Duc Trung P et al Excess body weight and age associated with the carriage of fluoroquinolone and third-generation cephalosporin resistance genes in commensal *Escherichia coli* from a cohort of urban Vietnamese children. J Med Microbiol 2018; 67: 1457–66. 30113307 10.1099/jmm.0.000820

[145] Thuy DB , Campbell J, Nhat LTH et al Hospital-acquired colonization and infections in a Vietnamese intensive care unit. PLoS One 2018; 13: e0203600. 30192894 10.1371/journal.pone.0203600 PMC6128614

[146] UNSD . Methodology. https://unstats.un.org/unsd/methodology/m49/.

