# Supplementary material for: Comparison of the global prevalence and trend of human intestinal carriage of ESBL-producing Escherichia coli between healthcare and community settings: a systematic review and meta-analysis
Source: JAC Antimicrob Resist. 2022 Jun 2;4(3):dlac048. doi: 10.1093/jacamr/dlac048 (PMC9160884; doi:10.1093/jacamr/dlac048)
Supplement: dlac048_Supplementary_Data [file dlac048_supplementary_data.docx]

**Supplementary data**

**Table S1:** PRISMA Checklist

**Table S2:** Summary table of 133 articles that that assessed the prevalence of faecal ESBL *E. coli* carriage either in healthy persons or inpatients worldwide, 2000-2021.

**Table S3:** Quality assessment criteria for the eligible studies

**Figure S1:** The global pooled prevalence in human intestinal carriage of ESBL *E. coli* in community versus healthcare settings. Abbreviations: *E. coli, Escherichia coli*.

**Figure S2:** The prevalence of human faecal ESBL *E.coli* carriage among the six WHO regions^21^ in community setting. Abbreviations: *E. coli, Escherichia coli*.

**Figure S3:** The prevalence of human faecal ESBL *E.coli* carriage among the six WHO regions^21^ in healthcare setting. Abbreviations: *E. coli, Escherichia coli*.

**Figure S4:** The global trend in the prevalence of human faecal ESBL *E. coli* carriage in the community setting by subgrouping studies every five years of study period.

**Figure S5:** The global trend in the prevalence of human faecal ESBL *E. coli* carriage in the healthcare setting by subgrouping studies every five years of study period

**Figure S6:** The prevalence of human faecal ESBL *E. coli* carriage by amount (in time) of contact to healthcare settings in Europe. Abbreviations: *E. coli, Escherichia coli*.

**Figure S7:** The prevalence of human faecal ESBL *E. coli* carriage in South Americas by study setting. Abbreviations: *E. coli, Escherichia coli*.

**Figure S8:** The prevalence of human faecal ESBL *E. coli* carriage in North Americas by study setting. Abbreviations: *E. coli, Escherichia coli*.

**Figures S9-S13:** Sub-meta-analysis results by sub-regions of Europe and the West Pacific

**Figure S14:** Funnel plots

**Table S1: PRISMA 2020 Checklist**

| **Section and Topic** | **Item #** | **Checklist item** | **Location where item is reported** |
| --- | --- | --- | --- |
| **TITLE** | | |  |
| Title | 1 | Identify the report as a systematic review. | p2 |
| **ABSTRACT** | | |  |
| Abstract | 2 | See the PRISMA 2020 for Abstracts checklist. | P2 and 3 |
| **INTRODUCTION** | | |  |
| Rationale | 3 | Describe the rationale for the review in the context of existing knowledge. | P4 |
| Objectives | 4 | Provide an explicit statement of the objective(s) or question(s) the review addresses. | P4 |
| **METHODS** | | |  |
| Eligibility criteria | 5 | Specify the inclusion and exclusion criteria for the review and how studies were grouped for the syntheses. | P5-8 |
| Information sources | 6 | Specify all databases, registers, websites, organisations, reference lists and other sources searched or consulted to identify studies. Specify the date when each source was last searched or consulted. | P5-8 |
| Search strategy | 7 | Present the full search strategies for all databases, registers and websites, including any filters and limits used. | P5-8 |
| Selection process | 8 | Specify the methods used to decide whether a study met the inclusion criteria of the review, including how many reviewers screened each record and each report retrieved, whether they worked independently, and if applicable, details of automation tools used in the process. | P5-8 |
| Data collection process | 9 | Specify the methods used to collect data from reports, including how many reviewers collected data from each report, whether they worked independently, any processes for obtaining or confirming data from study investigators, and if applicable, details of automation tools used in the process. | P5-8 |
| Data items | 10a | List and define all outcomes for which data were sought. Specify whether all results that were compatible with each outcome domain in each study were sought (e.g. for all measures, time points, analyses), and if not, the methods used to decide which results to collect. | P8 |
|  | 10b | List and define all other variables for which data were sought (e.g. participant and intervention characteristics, funding sources). Describe any assumptions made about any missing or unclear information. | P5-8 |
| Study risk of bias assessment | 11 | Specify the methods used to assess risk of bias in the included studies, including details of the tool(s) used, how many reviewers assessed each study and whether they worked independently, and if applicable, details of automation tools used in the process. | P8-9 |
| Effect measures | 12 | Specify for each outcome the effect measure(s) (e.g. risk ratio, mean difference) used in the synthesis or presentation of results. | P8-9 |
| Synthesis methods | 13a | Describe the processes used to decide which studies were eligible for each synthesis (e.g. tabulating the study intervention characteristics and comparing against the planned groups for each synthesis (item #5)). | P8-9 |
|  | 13b | Describe any methods required to prepare the data for presentation or synthesis, such as handling of missing summary statistics, or data conversions. | P8-9 |
|  | 13c | Describe any methods used to tabulate or visually display results of individual studies and syntheses. | P8-9 |
|  | 13d | Describe any methods used to synthesize results and provide a rationale for the choice(s). If meta-analysis was performed, describe the model(s), method(s) to identify the presence and extent of statistical heterogeneity, and software package(s) used. | P8-9 |
|  | 13e | Describe any methods used to explore possible causes of heterogeneity among study results (e.g. subgroup analysis, meta-regression). | P8-9 |
|  | 13f | Describe any sensitivity analyses conducted to assess robustness of the synthesized results. |  |
| Reporting bias assessment | 14 | Describe any methods used to assess risk of bias due to missing results in a synthesis (arising from reporting biases). | P8-9 |
| Certainty assessment | 15 | Describe any methods used to assess certainty (or confidence) in the body of evidence for an outcome. | P8-9 |
| **RESULTS** | | |  |
| Study selection | 16a | Describe the results of the search and selection process, from the number of records identified in the search to the number of studies included in the review, ideally using a flow diagram. | P9-10 |
|  | 16b | Cite studies that might appear to meet the inclusion criteria, but which were excluded, and explain why they were excluded. | P8-9 |
| Study characteristics | 17 | Cite each included study and present its characteristics. | P9-10, Table S2 |
| Risk of bias in studies | 18 | Present assessments of risk of bias for each included study. | P8-9 |
| Results of individual studies | 19 | For all outcomes, present, for each study: (a) summary statistics for each group (where appropriate) and (b) an effect estimate and its precision (e.g. confidence/credible interval), ideally using structured tables or plots. | P9-15, FiguresS1-S8 |
| Results of syntheses | 20a | For each synthesis, briefly summarise the characteristics and risk of bias among contributing studies. | P8-9 |
|  | 20b | Present results of all statistical syntheses conducted. If meta-analysis was done, present for each the summary estimate and its precision (e.g. confidence/credible interval) and measures of statistical heterogeneity. If comparing groups, describe the direction of the effect. | P9-15, FiguresS1-S8 |
|  | 20c | Present results of all investigations of possible causes of heterogeneity among study results. | P8-9, FiguresS1-S8 |
|  | 20d | Present results of all sensitivity analyses conducted to assess the robustness of the synthesized results. |  |
| Reporting biases | 21 | Present assessments of risk of bias due to missing results (arising from reporting biases) for each synthesis assessed. | P8-9 |
| Certainty of evidence | 22 | Present assessments of certainty (or confidence) in the body of evidence for each outcome assessed. | P9-15, FiguresS1-S8 |
| **DISCUSSION** | | |  |
| Discussion | 23a | Provide a general interpretation of the results in the context of other evidence. | P15-18 |
|  | 23b | Discuss any limitations of the evidence included in the review. | P15-18 |
|  | 23c | Discuss any limitations of the review processes used. | P17 |
|  | 23d | Discuss implications of the results for practice, policy, and future research. | P18 |
| **OTHER INFORMATION** | | |  |
| Registration and protocol | 24a | Provide registration information for the review, including register name and registration number, or state that the review was not registered. | NA |
|  | 24b | Indicate where the review protocol can be accessed, or state that a protocol was not prepared. |  |
|  | 24c | Describe and explain any amendments to information provided at registration or in the protocol. |  |
| Support | 25 | Describe sources of financial or non-financial support for the review, and the role of the funders or sponsors in the review. | P18 |
| Competing interests | 26 | Declare any competing interests of review authors. | P18 |
| Availability of data, code and other materials | 27 | Report which of the following are publicly available and where they can be found: template data collection forms; data extracted from included studies; data used for all analyses; analytic code; any other materials used in the review. | P18 |

*From:*  Page MJ, McKenzie JE, Bossuyt PM, Boutron I, Hoffmann TC, Mulrow CD, et al. The PRISMA 2020 statement: an updated guideline for reporting systematic reviews. BMJ 2021;372:n71. doi: 10.1136/bmj.n71

**Table S2:** Summary table of 133 articles that that assessed the prevalence of faecal ESBL *E. coli* carriage either in healthy persons or inpatients worldwide, 2000-2021.

| WHO area | Study name | Country | Year of study | Average (approximated) year of study | Study design | Study setting | Healthcare contact | Total number of individuals screened (stool sample) | Number of ESBL E. coli positive individuals among screened | Faecal ESBL E. coli carriage rate in (%) | Method of ESBL detection (stool sample) Screening, confirmatory | Quality score |
| --- | --- | --- | --- | --- | --- | --- | --- | --- | --- | --- | --- | --- |
| Africa | Medboua-Benbalagh et al., 2017 ^29^ | Algeria | 2012-2013 | 2013 | Cross-Sectional | Healthcare | admitted 48 hours | 171 | 93 | 54.4% | MacConkey with 4ug/ml ml of either cefotaxime or ceftazidimeDDST, PCR | Good |
| Africa | Ouedraogo et al., 2016 ^30^ | Burkina Faso | 2014 | 2014 | Prospective cohort | Healthcare | admitted | 113 | 39 | 34.5% | DDST, PCR | Good |
| Africa | Lonchel et al., 2013 ^31^ | Cameroon | 2009 | 2009 | Cross-sectional | Healthcare | admitted | 121 | 34 | 28.1% | DDST, PCR | Good |
| Africa | Mahamat et al., 2019 ^7^ | Chad | 2017 | 2017 | Cross-Sectional | Healthcare | admitted | 100 | 35 | 35.0% | ESBL agar plates (bioMérieux) DDST, PCR | Good |
| Africa | Aklilu et al., 2020 ^32^ | Ethiopia | 2018-2019 | 2019 | Cross-Sectional | Healthcare | admitted | 421 | 62 | 14.7% | DDST | Good |
| Africa | Desta et al., 2016 ^33^ | Ethiopia | 2012 | 2012 | Cross-Sectional | Healthcare | admitted | 267 | 95 | 35.6% | Chrome agar, Vitek 2 system (bioMérieux, France). | Good |
| Africa | Falgenhauer et al., 2019 ^34^ | Ghana | 2015 | 2015 | Prospective cohort | Healthcare | admitted | 54 | 33 | 61.1% | MacConkey agar with 1 mg/L ceftazidime and 1 mg/L cefotaxime, DDST, WGS | Good |
| Africa | Isendahl et al., 2012 ^35^ | Guinea-Bissau | 2010 | 2010 | Cross-Sectional | Healthcare | admitted 48 hours | 408 | 133 | 32.6% | VITEK2, PCR | Good |
| Africa | Rakotomalala et al., 2019 ^36^ | Madagascar | 2017-2018 | 2018 | Cross-Sectional | Healthcare | admitted 48 hours | 46 | 8 | 17.4% | Phenotypic, DDST | Good |
| Africa | Ben Sallem et al., 2014 ^37^ | Mauritanian Hospital | 2009 | 2009 | Cross-sectional | Healthcare | admitted | 87 | 5 | 5.7% | MacConkey agar with 2 ulg/mL cefotaxime, DDST, PCR | Good |
| Africa | Kurz et al., 2017 ^9^ | Rwanda | 2014 | 2014 | Prospective cohort | Healthcare | admitted | 392 | 134 | 34.2% | Chromogenic agar (Chromagar-ESBL, Mast Diagnostica, Germany), ESBL-AmpC-Detection Test, Mast Diagnostica (DDST I think) | Good |
| Africa | Founou et al., 2018 ^38^ | South Africa | 2017 | 2017 | Prospective cohort | Healthcare | admitted | 26 | 11 | 42.3% | ROSCO DIAGNOSTICA (Taastrup, Denmark, Vitek® 2 System, PCR) | Good |
| Africa | Lonchel et al., 2012 ^31^ | Cameroon | 2009 | 2009 | Cross-sectional | Community setting | Not applicable | 150 | 9 | 6.0% | Drigalski and MacConkey with cefotaxime (1.5 mg/L) and ceftazidime (2 mg/L), DDST, PCR | Good |
| Africa | Akinduti et al., 2018 ^39^ | Nigeria | 2016 | 2016 | Cross-sectional | Community setting | Not applicable | 406 | 14 | 3.4% | Starch-iodide paperacidometric method, double disc method, PCR | Fair |
| Africa | Ouedraogo et al., 2016 ^30^ | Burkina Faso | 2014 | 2014 | Cross-sectional | Community setting | Not applicable | 101 | 21 | 20.8% | Biomerieux ESBL agar plates (France), DDST, PCR | Good |
| Africa | Mahamat et al., 2019 ^7^ | Chad | 2017 | 2017 | Cross-sectional | Community setting | Not applicable | 100 | 29 | 29.0% | BioMérieux ESBL agar plates (France), double-disc synergy test, PCR | Good |
| Africa | Herindrainy et al., 2018 ^40^ | Madagascar | 2015-2016 | 2016 | Prospective cohort | Community setting | Not applicable | 275 | 28 | 10.2% | Chromagar ESBL (France), DDST | Good |
| Africa | Kurz et al., 2017 ^9^ | Rwanda | 2014 | 2014 | Prospective cohort | Community setting | Not applicable | 361 | 87 | 24.1% | Chromagar-ESBL Mast Diagnostica (Germany), ESBL-AmpC-Detection Test Mast Diagnostica | Good |
| Africa | Mshana et al., 2016 ^28^ | Tanzania | 2014 | 2014 | Cross-sectional | Community setting | Not applicable | 334 | 37 | 11.1% | Chromagar ESBL (France), VITEK-2 system, PCR | Good |
| Africa | Bu¨del et al., 2019 ^41^ | Tanzania | 2018 | 2018 | Cross-sectional | Community setting | Not applicable | 59 | 45 | 76.3% | Biomerieux ChromID ESBL (France), PCR | Good |
| America | Cort´es-Cort´es et al., 2017 ^42^ | Mexico | 2012 | 2012 | Cross-sectional | Community setting | Not applicable | 60 | 11 | 18.3% | Disk containig lactamaseinhibitr +PCR | Fair |
| America | Marusinec et al., 2021 ^43^ | Ecuador | 2018 | 2018 | Prospective cohort | Community setting | Not applicable | 374 | 44 | 11.8% | DDST | Good |
| America | Woerther et al.,2013 ^44^ | Guyana | 2010 | 2010 | Cross-sectional | Community setting | Not applicable | 151 | 8 | 5.3% | Chemunex ESBL plates (France), DDST, PCR | Good |
| America | Islam et al., 2019 ^45^ | USA | 2013-2015 | 2014 | Prospective cohort | Community setting | Not applicable | 519 | 18 | 3.5% | ESBL Chromagar plates (France), DDST | Good |
| America | Weisenberget al, 2012 ^46^ | USA | 2009-2010 | 2010 | Prospective cohort | Community setting | Not applicable | 60 | 1 | 1.7% | MacConkey with cefpodoxime (4ug/ml), DDST, PCR | Good |
| America | Araque, 2018 ^47^ | Venezuela | 2015 | 2015 | Cross-sectional | Community setting | Not applicable | 78 | 27 | 34.6% | MacConkey agar plates with cefotaxime (2mg/L), VITEK 2 system,PCR | Good |
| Americas | Vasques et al., 2010 ^48^ | Brazil | 2007 | 2007 | Cross-Sectional | Healthcare | ICU | 41 | 9 | 22.0% | Phenotypic, disk diffusion, PCR | Good |
| Americas | Harris et al., 2007 ^49^ | USA | 2001-2004 | 2003 | Prospective cohort | Healthcare | ICU | 1806 | 94 | 5.2% | MacConkey agar with 1 mg/mL of ceftazidime, disk diffusion, PFGE | Good |
| Americas | Han et al., 2012 ^50^ | USA | 2007-2009 | 2008 | Cross- sectional | Healthcare | admitted | 389 | 4 | 1.0% | DDST, PCR | Good |
| Eastern Mediterranean | Hashemizadeh et al., 2018 ^51^ | Iran | 2014-2015 | 2015 | Cross- sectional | Community setting | Not applicable | 100 | 48 | 48.0% | DDST, PCR | Good |
| Eastern Mediterranean | Rahman and El-Sherif, 2011 ^52^ | Egypt | 2010-2011 | 2011 | Cross- sectional | Community setting | Not applicable | 632 | 285 | 45.1% | MacConkey with 1ug/ml of cefotaxime, DDST | Good |
| Eastern Mediterranean | Fam et al., 2015 ^53^ | Egypt | 2013 | 2013 | Prospective cohort | Healthcare | admitted 48 hours | 58 | 44 | 75.9% | Drigalski agar with 0·5 mg/l cefotaxime,DDST, PCR | Good |
| Eastern Mediterranean | Aghamohammada et al., 2018 ^54^ | Iran | 2016 | 2016 | Cross-sectional | Healthcare | ICU | 61 | 32 | 52.5% | MacConkey agar with cefotaxime (1 mg/L) , DDST, PCR | Good |
| Eastern Mediterranean | Hashemizadeha et al., 2019 ^55^ | Iran | 2018 | 2018 | Cross-Sectional | Healthcare | admitted | 70 | 38 | 54.3% | DDST, PCR | Good |
| Eastern Mediterranean | Moubareck et al., 2005 ^56^ | Lebanon | 2003 | 2003 | Prospective cohort | Community setting | Not applicable | 382 | 9 | 2.4% | DDST, PCR | Good |
| Eastern Mediterranean | Hijazi et al., 2016 ^57^ | Lebanon | 2013 | 2013 | Cross- sectional | Community setting | Not applicable | 117 | 45 | 38.5% | DDST, PCR | Good |
| Eastern Mediterranean | Daoud et al., 2006 ^58^ | Lebanon | 2003 | 2003 | Prospective cohort | Healthcare | admitted | 378 | 58 | 15.3% | DDST | Good |
| Eastern Mediterranean | Barguigua et al., 2015 ^59^ | Morocco | 2013 | 2013 | Cross- sectional | Community setting | Not applicable | 93 | 1 | 1.1% | MacConkey (cefotaxime or ceftazidime 1mg/L), DDST | Good |
| Eastern Mediterranean | Kader and Kamath, 2009 ^60^ | Saudi Arabia | 2006-2007 | 2007 | Cross- sectional | Community setting | Not applicable | 505 | 62 | 12.3% | MacConkey (cefotaxime or ceftazidime 1ug/ml), DDST | Good |
| Eastern Mediterranean | Elkersh et al., 2015 ^61^ | Saudi Arabia | 2012-2013 | 2013 | Cross- sectional | Community setting | Not applicable | 150 | 22 | 14.7% | Disc diffusion(screening), DDST | Good |
| Eastern Mediterranean | Kader et al., 2007 ^62^ | Saudi Arabia | 2006 | 2006 | Prospective cohort | Healthcare | admitted | 144 | 46 | 31.9% | MacConkey agar with 1 ug/mL cefotaxime or ceftazidime, DDST | Good |
| Eastern Mediterranean | Sana et al., 2016 ^63^ | Tunisia | 2013 | 2013 | Cross- sectional | Community setting | Not applicable | 105 | 7 | 6.7% | Cefotaxime (2µg/ml), DDST, PCR | Fair |
| Europe | Valenza et al., 2013 ^64^ | Germany | 2009-2012 | 2011 | Prospective cohort | Community setting | Not applicable | 3344 | 211 | 6.3% | MacConkey agar with 1mg/L cefotaxime, DDST, PCR | Good |
| Europe | Lübbert et al., 2015 ^65^ | Germany | 2013 | 2013 | Prospective cohort | Community setting | Not applicable | 205 | 14 | 6.8% | BioMérieux ChromagarTM ESBL (Paris, France), Etest (bioMérieux, Marcy l’Etoile, France), PCR | Good |
| Europe | Guimaraes et al., 2009 ^66^ | Portugal | 2007-2008 | 2008 | Cross- sectional | Community setting | Not applicable | 112 | 3 | 2.7% | Levine agar plates with 2mg/L cefotaxime, DDST, PCR | Good |
| Europe | Latour et al., 2019 ^67^ | Belgium | 2015 | 2015 | Cross-Sectional | Healthcare | Nursing care | 1423 | 143 | 10.0% | DDST, PCR | Good |
| Europe | Ruh et al., 2019 ^68^ | Cyprus | 2017 | 2017 | Cross-sectional | Community setting | Not applicable | 500 | 101 | 20.2% | MacConkey agar with cefotaxime and ceftazidime 1mg/L, DDST | Good |
| Europe | Dall et al., 2019 ^69^ | Denmark | 2014-2017 | 2016 | Prospective cohort | Community setting | Not applicable | 50 | 6 | 12.0% | Biomerieux ChromID ESBL (France), ESBL + AmpC Screen Kit (Rosco, Denmark) | Good |
| Europe | Chanoine et al., 2012 ^70^ | France | 2011 | 2011 | Cross-sectional | Community setting | Not applicable | 345 | 21 | 6.1% | Biomerieux Chromogenic ESBL agar plates (France), DDST, PCR | Good |
| Europe | Guibout et al., 2008 ^71^ | France | 2006 | 2006 | Cross-sectional | Community setting | Not applicable | 322 | 2 | 0.6% | Drigalski agar plates with 0.5mg/L cefotaxime, PCR | Good |
| Europe | Janvier et al., 2011 ^72^ | France | 2009 | 2009 | Prospective cohort | Community setting | Not applicable | 512 | 22 | 4.3% | DDST, PCR | Good |
| Europe | Pilmis et al., 2018 ^73^ | France | 2014 | 2014 | Cross-sectional | Healthcare | admitted | 554 | 97 | 17.5% | ChromID ESBL, bioMérieux, Marcy-l'Etoile,France; combination disc method | Good |
| Europe | Navarro et al., 2010 ^74^ | France | 2009 | 2009 | Prospective cohort | Healthcare | admitted | 303 | 32 | 10.6% | Drigalski agar with, ceftazidime, ESBL Chromagar, DDST | |
| Europe | Boutet-Dubois et al., 2013 ^75^ | France | 2010-2011 | 2011 | Prospective cohort | Healthcare | admitted 48 hours | 1118 | 58 | 5.2% | Drigalski agar with ceftazidime,ESBL Chromagar® media, Rosco tablets test, PCR | Good |
| Europe | Bastard et al., 2020 ^76^ | France | 2019 | 2019 | Cross-Sectional | Healthcare | Nursing care | 144 | 7 | 4.9% | Method not written | Good |
| Europe | Jolivet et al., 2018 ^77^ | France | 2016 | 2016 | Cross-Sectional | Healthcare | admitted | 844 | 98 | 11.6% | ChromID ESBL (bioMérieux),DDST, PCR | Good |
| Europe | Meyer et al., 2012 ^78^ | Germany | 2011 | 2011 | Cross- sectional | Community setting | Not applicable | 231 | 8 | 3.5% | ChromID ESBL screening, VITEK 2 system. | Good |
| Europe | Vehreschild et al., 2014 ^79^ | Germany | 2011-2012 | 2012 | Prospective cohort | Healthcare | admitted | 497 | 49 | 9.9% | Vitek 2 (bioMe´ rieux, Nu¨ rtingen, Germany) | Good |
| Europe | Hamprecht et al., 2016 ^80^ | Germany | 2014 | 2014 | Prospective cohort | Healthcare | admitted | 4376 | 344 | 7.9% | ChromID ESBL agar (Biomerieux, Nu¨ rtingen, Germany) DDST, PCR | Good |
| Europe | Reinheimer et al., 2016 ^81^ | Germany | 2014-2016 | 2015 | Cross-sectional | Healthcare | admitted | 218 | 25 | 11.5% | Chromagar ESBL plates, VITEK 2 | Good |
| Europe | Valenza et al., 2015 ^64^ | Germany | 2013-2014 | 2014 | Cross-Sectional | Healthcare | Nursing care | 156 | 23 | 14.7% | MacConkey with cefotaxime (1 mg/L), DDST | Good |
| Europe | Arvand et al., 2017 ^82^ | Germany | 2010-2011 | 2011 | Cross-Sectional | Healthcare | Nursing care | 305 | 23 | 7.5% | Brilliance ESBL agar (oxoid), PCR | Good |
| Europe | Ebrahimi et al., 2016 ^83^ | Hungary | 2013-2014 | 2014 | Cross-sectional | Community setting | Not applicable | 779 | 18 | 2.3% | EMB with 2 mg/L cefotaxime, DDST, PCR | Good |
| Europe | Ebrahimi et al., 2016 ^84^ | Hungary | 2010-2013 | 2012 | Prospective cohort | Healthcare | admitted | 4343 | 323 | 7.4% | EMB with 2 mg/L cefotaxime, double disk synergy test | Good |
| Europe | Adler et al., 2012 ^85^ | Israel | 2008-2009 | 2009 | Prospective cohort | Healthcare | admitted | 492 | 125 | 25.4% | Brilliance ESBL Agar (Oxoid,Basingstoke, UK), DDST, PCR | Good |
| Europe | Giufre et al., 2017 ^86^ | Italy | 2015 | 2015 | Cross-sectional | Healthcare | Nursing care | 487 | 239 | 49.1% | Double-disc synergy testing, PCR | Good |
| Europe | March et al., 2009 ^87^ | Italy | 2008 | 2008 | Cross-sectional | Healthcare | Nursing care | 111 | 41 | 36.9% | Vitek 2 System | Good |
| Europe | Meletiadis et al., 2017 ^88^ | Italy, Serbia, Romania | 2007-2013 | 2010 | Prospective cohort | Healthcare | admitted 48 hours | 10035 | 1102 | 11.0% |  | Fair |
| Europe | Willemsen, et al., 2015 ^89^ | Netherlands | 2012 | 2012 | Cross- sectional | Healthcare | Nursing care | 160 | 32 | 20.0% | DDST, PCR | Good |
| Europe | Bunt et al., 2019 ^10^ | Netherlands | 2014-2016 | 2015 | Cross- sectional | Community setting | Not applicable | 4177 | 174 | 4.2% | MacConkey agar with 1 mg/L cefotaxime, PCR | Good |
| Europe | Overdevest et al., 2016 ^90^ | Netherlands | 2013-2014 | 2014 | Prospective cohort | Healthcare | Nursing care | 296 | 69 | 23.3% | DDST, PCR | Good |
| Europe | Bergh et al., 2017 ^91^ | Netherlands | 2011-2014 | 2013 | Multi-center cluster-randomized studies | Healthcare | admitted | 2797 | 223 | 8.0% | EbSA (Cepheid Benelux, Apeldoorn, the Netherlands), ChromID ESBL (bioMérieux, Marcy l’Etoile, France), DDST, Etest | Good |
| Europe | Ulstad et al., 2016 ^92^ | Norway | 2014-2016 | 2015 | Cross- sectional | Community setting | Not applicable | 308 | 14 | 4.5% | MacConkey agar plates with cefotaxime (1 mg/L), Total ESBL + AmpC Confirm kit (Rosco Diagnostica, Denmark), PCR | Good |
| Europe | Sadowska-Klasa et al., 2017 ^93^ | Poland, | 2012-2014 | 2013 | Cross-sectional | Healthcare | admitted | 120 | 32 | 26.7% | Not mentioned | Fair |
| Europe | Aires-de-Sousa et al., 2019 ^94^ | Portugal | 2018-2019 | 2019 | Cross-Sectional | Healthcare | admitted 48 hours | 151 | 14 | 9.3% | Chromagar ESBL (Frilabo,Maia, Portugal),disk diffusion, PCR | Good |
| Europe | Valverde et al., 2004 ^95^ | Spain | 2003 | 2003 | Cross-sectional | Community setting | Not applicable | 108 | 4 | 3.7% | MacConkey (cefotaxime or ceftazidime 1ug/ml), DDST, PCR | Good |
| Europe | Rámila et al., 2018 ^96^ | Spain | 2014-2015 | 2015 | Cross- sectional | Community setting | Not applicable | 815 | 57 | 7.0% | Combination disk test on Mueller Hinton with and without 250 mg/L cloxacillin, PCR | Fair |
| Europe | Paniagua et al., 2010 ^97^ | Spain | 2007 | 2007 | Prospective cohort | Healthcare | admitted | 146 | 13 | 8.9% | ceftazidime or cefotaxime (1 mg/L), ChromID ESBL, bioMérieux, Marcy-l'Etoile,France | Good |
| Europe | Valverde et al., 2004 ^95^ | Spain | 2003 | 2003 | Prospective cohort | Healthcare | admitted | 102 | 12 | 11.8% | MacConkey agar with 1 ug/mL ceftazidime and 1 ug/mL cefotaxime, DDST | Good |
| Europe | Calatayud et al., 2008 ^98^ | Spain | 2006-2007 | 2007 | Prospective cohort | Healthcare | admitted | 154 | 49 | 31.8% | MacConkey agar with cefotaxime (2ug/ml) and ceftazidime (4ug/ml), DDST, PCR | Good |
| Europe | Miro et al., 2005 ^99^ | Spain | 2001-2002 | 2002 | Cross-Sectional | Healthcare | admitted | 1321 | 44 | 3.3% | MacConkey agar with cefotaxime (2ug/ml) , DDST, PCR | Good |
| Europe | Colmenarejo et al., 2020 ^100^ | Spain | 2018 | 2018 | Cross- sectional | Healthcare | Nursing care | 187 | 55 | 29.4% | Brilliance ESBL agar(UK), DDST, PCR | Good |
| Europe | Kaarme et al., 2013 ^101^ | Sweden | 2010 | 2010 | Prospective cohort | Community setting | Not applicable | 313 | 8 | 2.6% | Luria-Bertani broth (USA) with cefpodoxime (5ug/mL), double disc approximation, PCR | Good |
| Europe | Kaarme et al., 2018 | Sweden | 2016 | 2016 | Prospective cohort | Community setting | Not applicable | 334 | 43 | 12.9% | Luria-Bertani broth (USA) with cefpodoxime (5ug/mL), double disc approximation, PCR | Good |
| Europe | Ny et al., 2016 ^102^ | Sweden | 2012-2013 | 2013 | Cross-sectional | Community setting | Not applicable | 2134 | 101 | 4.7% | Chromoriental-agar plate supplemented with 3 mg/L cefpodoxime+ PCR | Good |
| Europe | Strömdahl et al., 2011 ^103^ | Sweden | 2008-2010 | 2009 | Cross-Sectional | Healthcare | admitted | 231 | 10 | 4.3% | ChromID ESBL (bioMérieux),DDST, PCR | Good |
| Europe | Chabok et al., 2010 ^104^ | Sweden | 2006-2007 | 2007 | Cross-Sectional | Healthcare | admitted | 208 | 9 | 4.3% | Chromogenic agar, Etest, PCR | Good |
| Europe | Blom et al., 2016 ^105^ | Sweden | 2014 | 2014 | Cross-Sectional | Healthcare | Nursing care | 91 | 10 | 11.0% | ChromID ESBL (bioMérieux), DDST | Good |
| Europe | Andersson et al., 2012 ^106^ | Sweden | 2008 | 2008 | Prospective cohort | Healthcare | Nursing care | 495 | 14 | 2.8% | ChromID ESBL (bioMérieux), DDST, PCR | Good |
| Europe | Kuenzli et al., 2014 ^107^ | Switzerland | 2012-2013 | 2013 | Prospective cohort | Community setting | Not applicable | 179 | 5 | 2.8% | BioMérieux ChromID® ESBL (France), Vitek-2® system, PCR | Good |
| Europe | Tigen et al., 2014 ^108^ | Turkey | 2008-2010 | 2009 | Prospective cohort | Community setting | Not applicable | 400 | 75 | 18.8% | MacConkey with 1 mg/mL cefotaxime or 1mg/mL ceftazidime, Modified combined disk method, PCR | Good |
| Europe | Kizilates et al., 2020 ^14^ | Turkey | 2015 | 2015 | Cross- sectional | Healthcare | admitted <48 hours | 168 | 39 | 23.2% | Vitek-2 (bioMerieux), PCR | Good |
| Europe | Blane et al., 2016 ^109^ | UK | 2014 | 2014 | Prospective cohort | Healthcare | Nursing care | 37 | 15 | 40.5% | ChromID and Brilliance ESBL agars,MALDI-TOF MS,Vitek 2 instrument, PCR | Good |
| Europe | Munday et al., 2004 ^110^ | UK | 2003 | 2003 | Cross-Sectional | Healthcare | admitted | 394 | 1 | 0.3% | MacConkey agar with vancomycin (6 mg/L) and either cefpodoxime (4 mg/L), cefotaxime (1 mg/L) or ceftazidime (1 mg/L). DDST, PCR | Good |
| South east asia | Mathai et al., 2014 ^111^ | India | 2005-2007 | 2006 | Cross-sectional | Community setting | Not applicable | 115 | 22 | 19.1% | MacConkey with Ceftazidime (2ug/ul), DDST, PCR | Good |
| South east asia | Rousham et al., 2021 ^112^ | Bangladesh | 2017-2018 | 2018 | Cross- sectional | Community setting | Not applicable | 200 | 135 | 67.5% | Chromagar ESBL, PCR | Good |
| South east asia | Babu et al., 2016 ^113^ | India | 2011-2013 | 2012 | Prospective cohort | Community setting | Not applicable | 260 | 69 | 26.5% | MacConkey with 1ug/ml ceftazidime, PCR | Good |
| South east asia | Maharjan et al., 2018 ^114^ | Nepal | 2016 | 2016 | Cross-sectional | Community setting | Not applicable | 510 | 34 | 6.7% | DDST, PCR | Good |
| South east asia | Luvsansharav et al., 2012 ^115^ | Thailand | 2010 | 2010 | Cross-sectional | Community setting | Not applicable | 417 | 234 | 56.1% | MacConkey with cefotaxime 2 mg /ml, DDST, PCR | Good |
| South-East Asia | Babu et al., 2016 ^113^ | India | 2011-2013 | 2012 | Prospective cohort | Healthcare | admitted | 220 | 97 | 44.1% | MacConkey agar supplemented with ceftazidime | Good |
| South-East Asia | Mulki et al., 2017 ^116^ | India | 2016 | 2016 | Cross-Sectional | Healthcare | ICU | 60 | 22 | 36.7% | ChromID ESBL media, BioMerieux, France, DDST | Good |
| South-East Asia | Severin et al., 2012 ^117^ | Indonesia | 2001–2002 | 2002 | Cross-sectional | Healthcare | admitted | 999 | 95 | 9.5% | Chromagar(Becton Dickinson, Heidelberg, Germany), DDST, PCR | Good |
| South-East Asia | Kiddee et al., 2019 ^118^ | Thailand | 2014-2015 | 2015 | Prospective cohort | Healthcare | ICU | 215 | 90 | 41.9% | Chrome UTI agar (Oxoid, Basingstoke, United Kingdom) with vancomycin (25ug/mL) and cefotaxime(1ug/mL) LDDST, PCR | Good |
| Western Pacific Region | Tian et al., 2008 ^119^ | China | 2007* | 2007 | Cross-sectional | Community setting | Not applicable | 270 | 19 | 7.0% | EMB with 1ug cefotaxime, VITEK 2 system (bioMérieux, Marcy l’Etoile, France), PCR | Good |
| Western Pacific Region | Sadahira et al., 2017 ^120^ | Japan | 2013-2015 | 2014 | Prospective cohort | Community setting | Not applicable | 640 | 85 | 13.3% | ChromAgar/ESBL plates- Kanto Chemical (Tokyo, Japan) | Good |
| Western Pacific Region | Nakane et al., 2016 ^121^ | Japan | 2010-2011 | 2011 | Cross- sectional | Community setting | Not applicable | 2230 | 70 | 3.1% | DDST, PCR | Good |
| Western Pacific Region | Nakayama et al., 2015 ^122^ | Vietnam | 2013 | 2013 | Cross-sectional | Community setting | Not applicable | 198 | 93 | 47.0% | MacConkey with 1 mg/L cefotaxime, DDST, PCR | Good |
| Western Pacific Region | Kennedy & Collignon, 2010 ^123^ | Australia | 2008-2009 | 2009 | Prospective cohort | Community setting | Not applicable | 106 | 2 | 1.9% | chro mID ESBL (bioMérieux, France), PCR | Good |
| Western Pacific Region | Stuart et al., 2011 ^124^ | Australia | 2010 | 2010 | Cross-Sectional | Healthcare | Nursing care | 119 | 14 | 11.8% | ChromID ESBL (bioMérieux),DDST, PCR | Good |
| Western Pacific Region | Lim et al., 2014 ^125^ | Australia | 2011 | 2011 | Cross-Sectional | Healthcare | Nursing care | 115 | 12 | 10.4% | ChromID ESBL (bioMérieux), DDST, PCR | Good |
| Western Pacific Region | Atterby et al., 2019 ^126^ | Cambodia | 2011 | 2011 | Cross- sectional | Community setting | Not applicable | 307 | 53 | 17.3% | ChromID OXA-48 (BioMérieux), ChromID CARBA (BioMérieux) and C3GR Chromag), PCR | Good |
| Western Pacific Region | Zhou et al., 2015 ^127^ | China | 2012 | 2012 | Cross- sectional | Community setting | Not applicable | 200 | 117 | 58.5% | ChromID ESBL agar (Biomerieux, Marcy l’Etoile, France), DDST, PCR | Good |
| Western Pacific Region | Qin et al., 2013 ^128^ | China | 2008-2011 | 2010 | Prospective cohort | Community setting | Not applicable | 41 | 3 | 7.3% | ESBL scre, Disk diffusion, PCR | Good |
| Western Pacific Region | Ni et al., 2011 ^129^ | China | 2009 | 2009 | Cross- sectional | Community setting | Not applicable | 109 | 55 | 50.5% | MacConkey with 1 mg/L cefotaxime or 1 mg/L ceftazidime, PCR | Good |
| Western Pacific Region | Ni et al., 2016 ^130^ | China | 2014 | 2014 | Cross-sectional | Community setting | Not applicable | 1732 | 528 | 30.5% | MacConkey with 4 µg/ml cefotaxime, Chromagar ESBL plates (Mei xiang, China), PCR | Fair |
| Western Pacific Region | Xu et al., 2017 ^131^ | China | 2012 | 2012 | Retrospective cohort | Healthcare | admitted | 650 | 167 | 25.7% | ChromID ESBL agar, Vitek 2 automatic system, pcr | Good |
| Western Pacific Region | Wen et al., 2010 ^132^ | China | 2007-2008 | 2008 | Prospective intervention study (non-controlled) | Healthcare | admitted | 296 | 119 | 40.2% | DDST | Good |
| Western Pacific Region | Kamei et al., 2017 ^133^ | Japan | 2013-2015 | 2014 | Prospective cohort | Community setting | Not applicable | 379 | 22 | 5.8% | ChromID ESBL (Sysmex-bio-Merieux, Tokyo, Japan), PCR | Good |
| Western Pacific Region | Higa et al., 2018 ^134^ | Japan | 2017 | 2017 | Cross- sectional | Community setting | Not applicable | 263 | 31 | 11.8% | Mackonckey with 2ug/ml cefotaxime, DDST | Good |
| Western Pacific Region | Nakamura et al., 2016 ^135^ | Japan | 2011-2012 | 2012 | Prospective cohort | Community setting | Not applicable | 496 | 42 | 8.5% | MacConkey with 1 mg/L cefotaxime or 1 mg/L ceftazidime, DDST, PCR | Good |
| Western Pacific Region | Nakamura et al., 2015 ^135^ | Japan | 2011-2012 | 2012 | Prospective cohort | Healthcare | admitted | 257 | 32 | 12.5% | Double-disk synergy test (DDST), PCR | Good |
| Western Pacific Region | Luvsansharav et al., 2013 ^136^ | Japan | 2010 | 2010 | Cross-sectional | Healthcare | Nursing care | 225 | 41 | 18.2% | MacConkey agar with 2 mg/L cefotaxime or ceftazidime, DDST, PCR | Good |
| Western Pacific Region | Takano et al., 2018 ^137^ | Japan | 2016 | 2016 | Cross-Sectional | Healthcare | Nursing care | 146 | 38 | 26.0% | Neg EN Combo1J panel, DDST, PCR | |
| Western Pacific Region | Kawamura et al., 2018 ^138^ | Japan | 2015-2017 | 2016 | Cross-Sectional | Healthcare | Nursing care | 258 | 59 | 22.9% | Chromagar ESBL medium(Kanto Chemical Co.,Inc., Tokyo, Japan),PCR | Good |
| Western Pacific Region | Nakayama et al., 2015 ^122^ | Laos | 2012 | 2012 | Cross- sectional | Community setting | Not applicable | 57 | 40 | 70.2% | MacConkey with 2 mg/L cefotaxime, DDST, PCR | Good |
| Western Pacific Region | Balji et al., 2016 ^139^ | Mongolia | 2014 | 2014 | Cross-sectional | Healthcare | admitted | 1050 | 412 | 39.2% | (Chromagar ESBL, VITEK 2 sys tem | Good |
| Western Pacific Region | Mo et al., 2019 ^140^ | Singapore | 2016-2017 | 2017 | Cross- sectional | Community setting | Not applicable | 305 | 71 | 23.3% | Chromagar ESBL((bioMérieux), whole genome sequencing of all ESBL positive E. coli | Good |
| Western Pacific Region | Joo et al., 2018 ^141^ | South Korea | 2014 | 2014 | Cross-sectional | Community setting | Not applicable | 109 | 20 | 18.3% | PCR | Good |
| Western Pacific Region | Ko et al., 2013 ^8^ | South Korea | 2011 | 2011 | Cross-sectional | Healthcare | ICU | 94 | 22 | 23.4% | Chromogenic medium, Vitek 2 system, | Good |
| Western Pacific Region | Wu et al., 2019 ^142^ | Taiwan | 2016-2017 | 2017 | Cross- sectional | Community setting | Not applicable | 724 | 14 | 1.9% | Chromagar™,ESBL plate (Chromagar Paris, France), PCR | Good |
| Western Pacific Region | Huang et al., 2020 ^143^ | Taiwan | 2017-2018 | 2018 | Cross- sectional | Community setting | Not applicable | 187 | 74 | 39.6% | ChromID ESBL agar (BioMerieux), PCR | Good |
| Western Pacific Region | Nhi et al., 2018 ^144^ | Vietnam | 2016 | 2016 | Prospective cohort | Community setting | Not applicable | 498 | 374 | 75.1% | MacConkey with ceftriaxone 6mg/L, PCR | Good |
| Western Pacific Region | Thuy et al., 2018 ^145^ | Vietnam | 2014-2016 | 2015 | Prospective cohort | Healthcare | ICU | 364 | 124 | 34.1% | Chromagar (Chromagar, Paris, France), DDST | Fair |

**Table S3:** Quality assessment criteria for the eligible studies

| **Criteria** | **Yes** | **No** | **Other (CD, NR, NA)*** |
| --- | --- | --- | --- |
| 1. Was the research question or objective in this paper clearly stated? |  |  |  |
| 2. Was the study population clearly specified and defined? |  |  |  |
| 3. Was the participation rate of eligible persons at least 50%? |  |  |  |
| 4. Were all the subjects selected or recruited from the same or similar populations (including the same time period)? Were inclusion and exclusion criteria for being in the study prespecified and applied uniformly to all participants? |  |  |  |
| 5. Was a sample size justification, power description, or variance and effect estimates provided? |  |  |  |
| 6. For the analyses in this paper, were the exposure(s) of interest measured prior to the outcome(s) being measured? |  |  |  |
| 7. Was the timeframe sufficient so that one could reasonably expect to see an association between exposure and outcome if it existed? |  |  |  |
| 8. For exposures that can vary in amount or level, did the study examine different levels of the exposure as related to the outcome (e.g., categories of exposure, or exposure measured as continuous variable)? |  |  |  |
| 9. Were the exposure measures (independent variables) clearly defined, valid, reliable, and implemented consistently across all study participants? |  |  |  |
| 10. Was the exposure(s) assessed more than once over time? |  |  |  |
| 11. Were the outcome measures (dependent variables) clearly defined, valid, reliable, and implemented consistently across all study participants? |  |  |  |
| 12. Were the outcome assessors blinded to the exposure status of participants? |  |  |  |
| 13. Was loss to follow-up after baseline 20% or less? |  |  |  |
| 14. Were key potential confounding variables measured and adjusted statistically for their impact on the relationship between exposure(s) and outcome(s)? |  |  |  |

| **Quality Rating (Good, Fair, or Poor)** |
| --- |
| Rater #1 initials: YB |
| Rater #2 initials: WB |
| Additional Comments (If POOR, please state why): |

*CD, cannot determine; NA, not applicable; NR, not reported


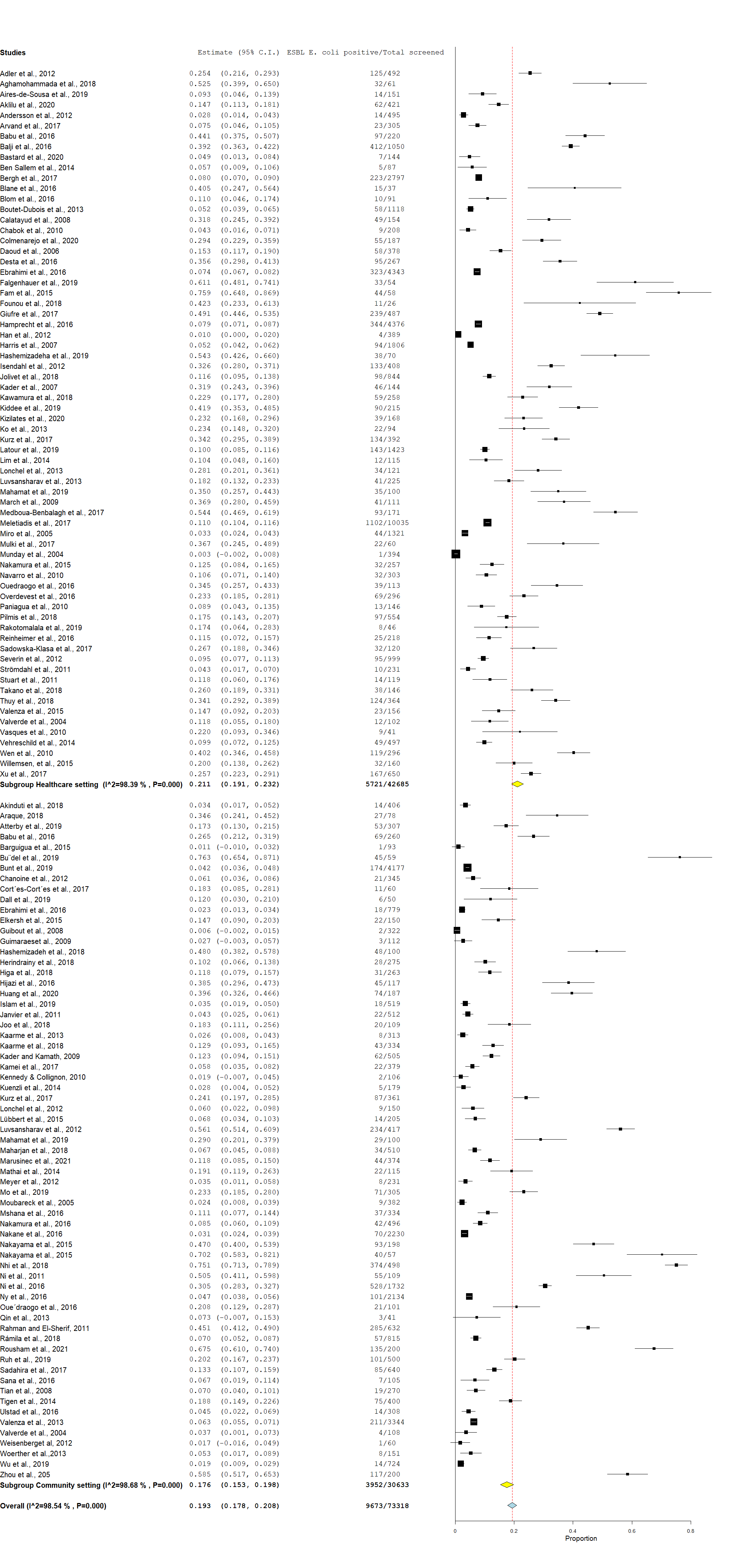


**Figure S1:** The global pooled prevalence in human intestinal carriage of ESBL *E. coli* in community versus healthcare settings.


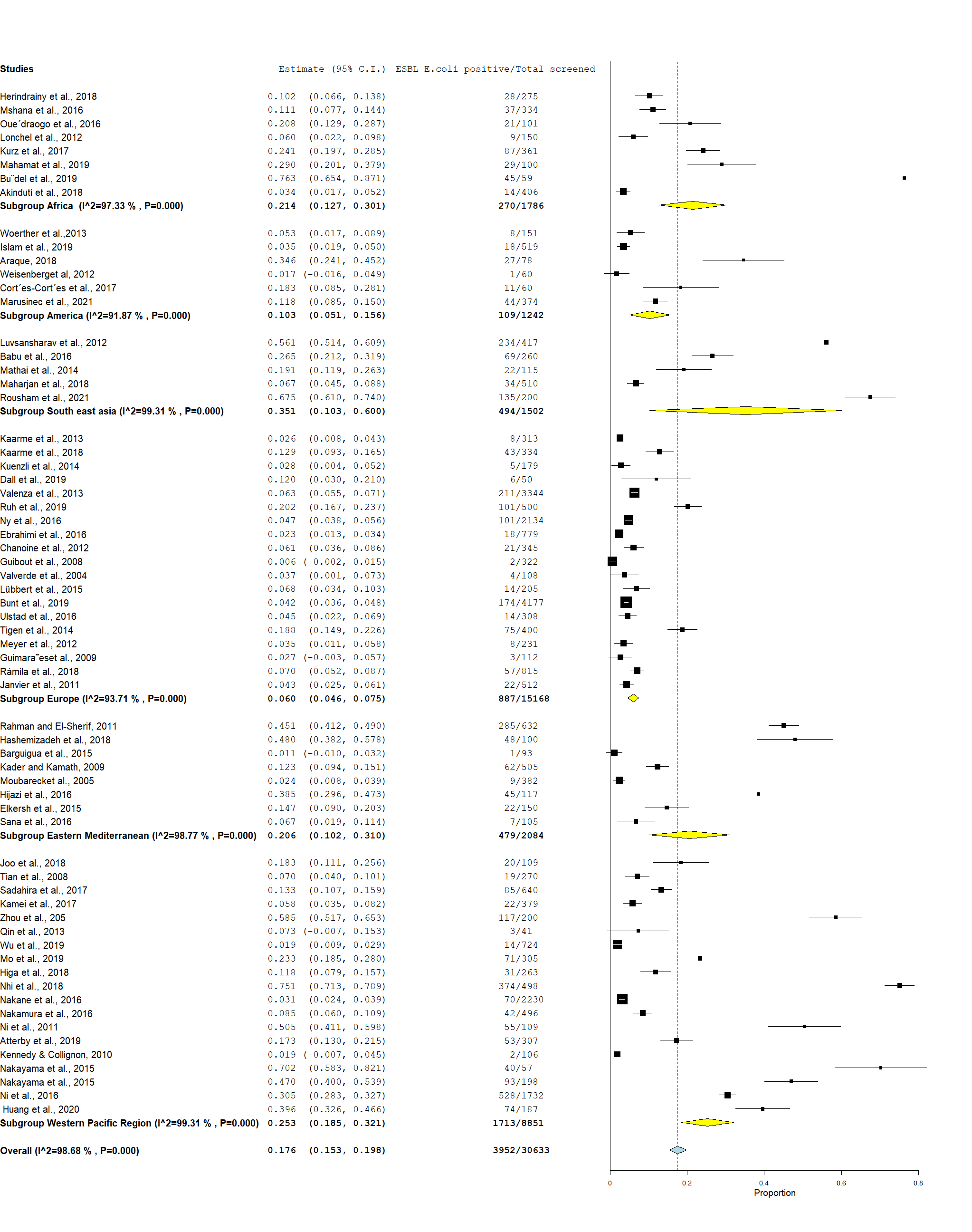


**Figure S2:** The prevalence of human faecal ESBL *E. coli* carriage among the six WHO regions^21^ in community settings.


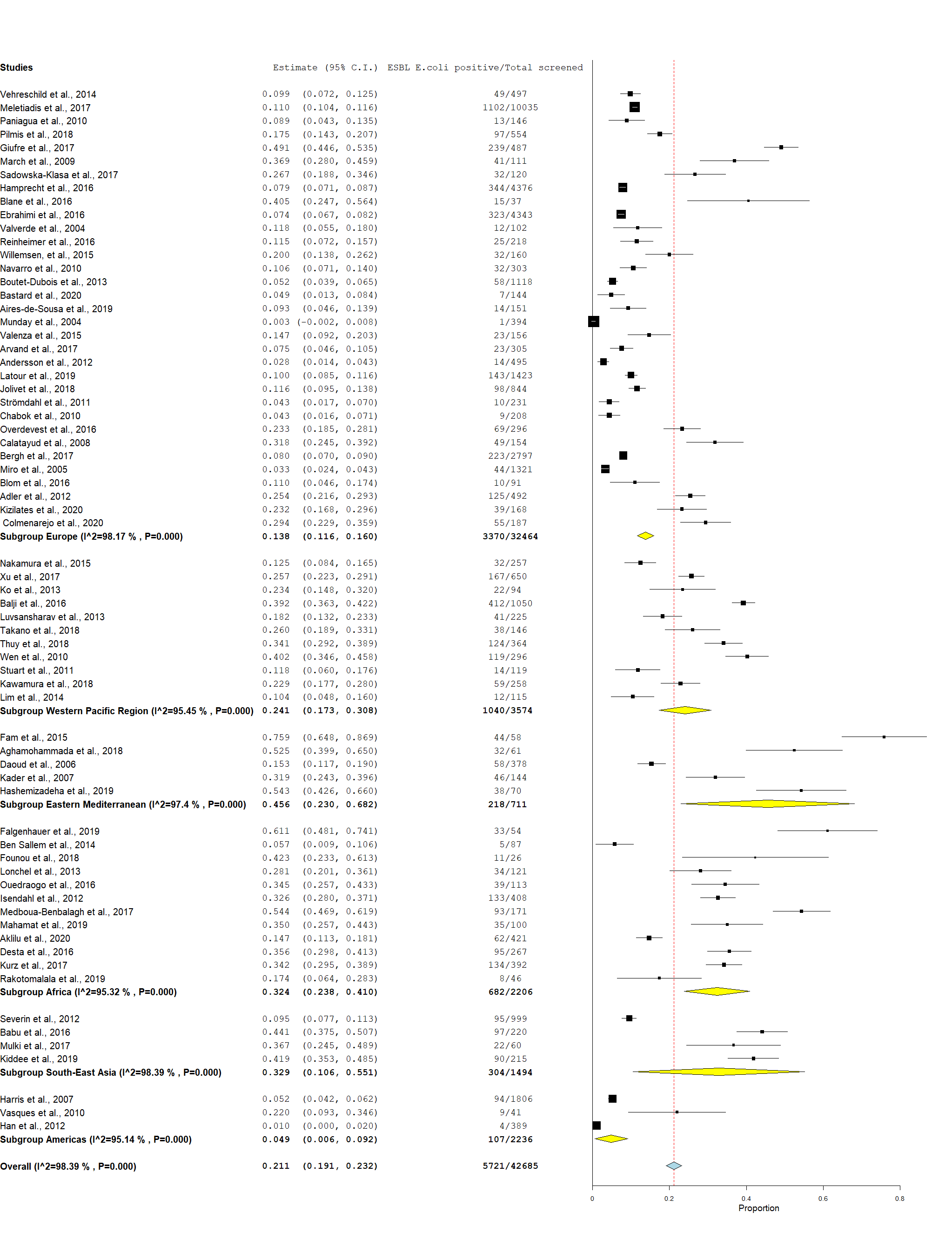


**Figure S3:** The prevalence of human faecal ESBL *E. coli* carriage among the six WHO regions^21^ in healthcare settings.


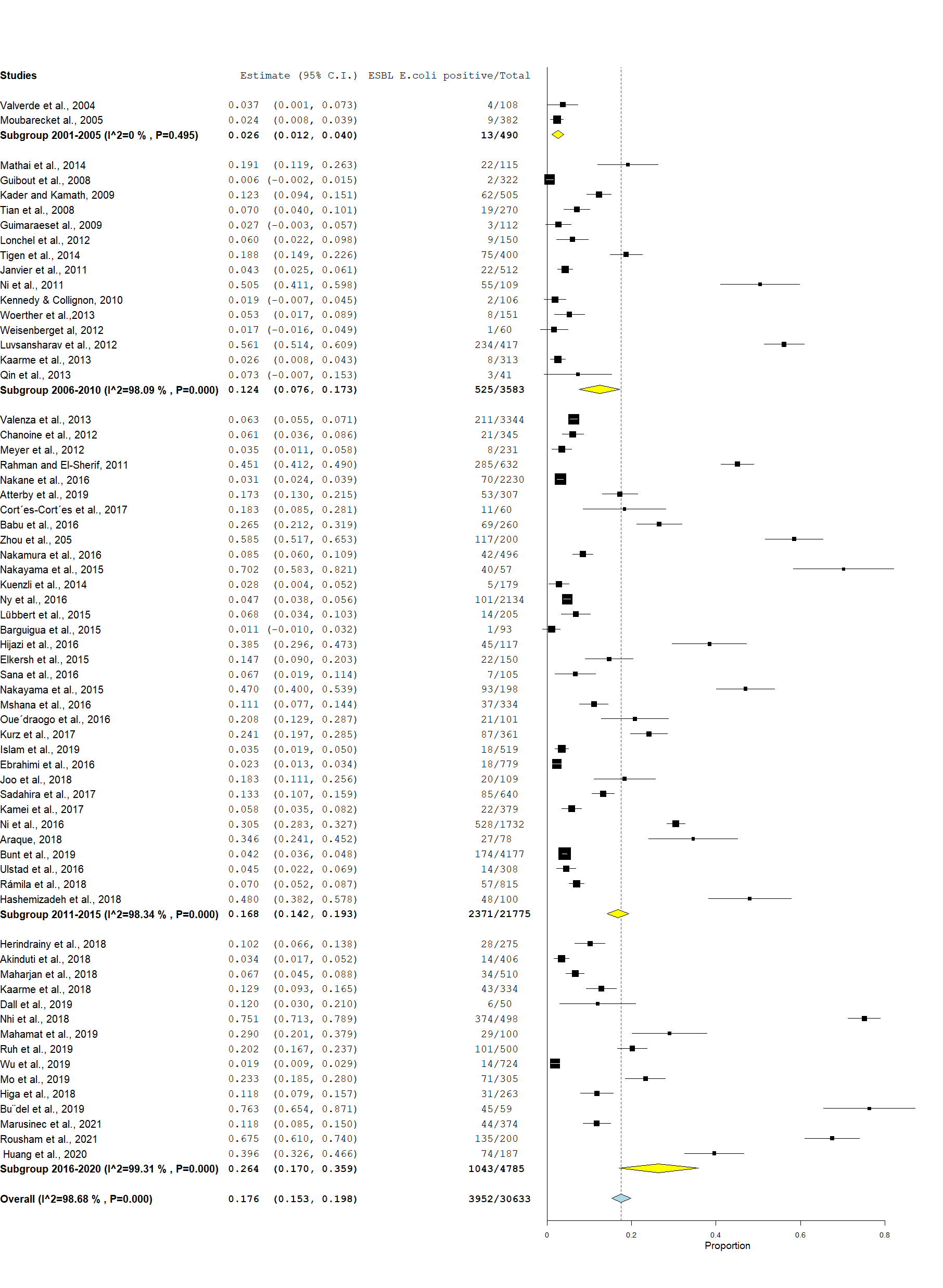


**Figure S4:** The global trend in the prevalence of human faecal ESBL *E. coli* carriage in the community setting by subgrouping studies every five years of study period.


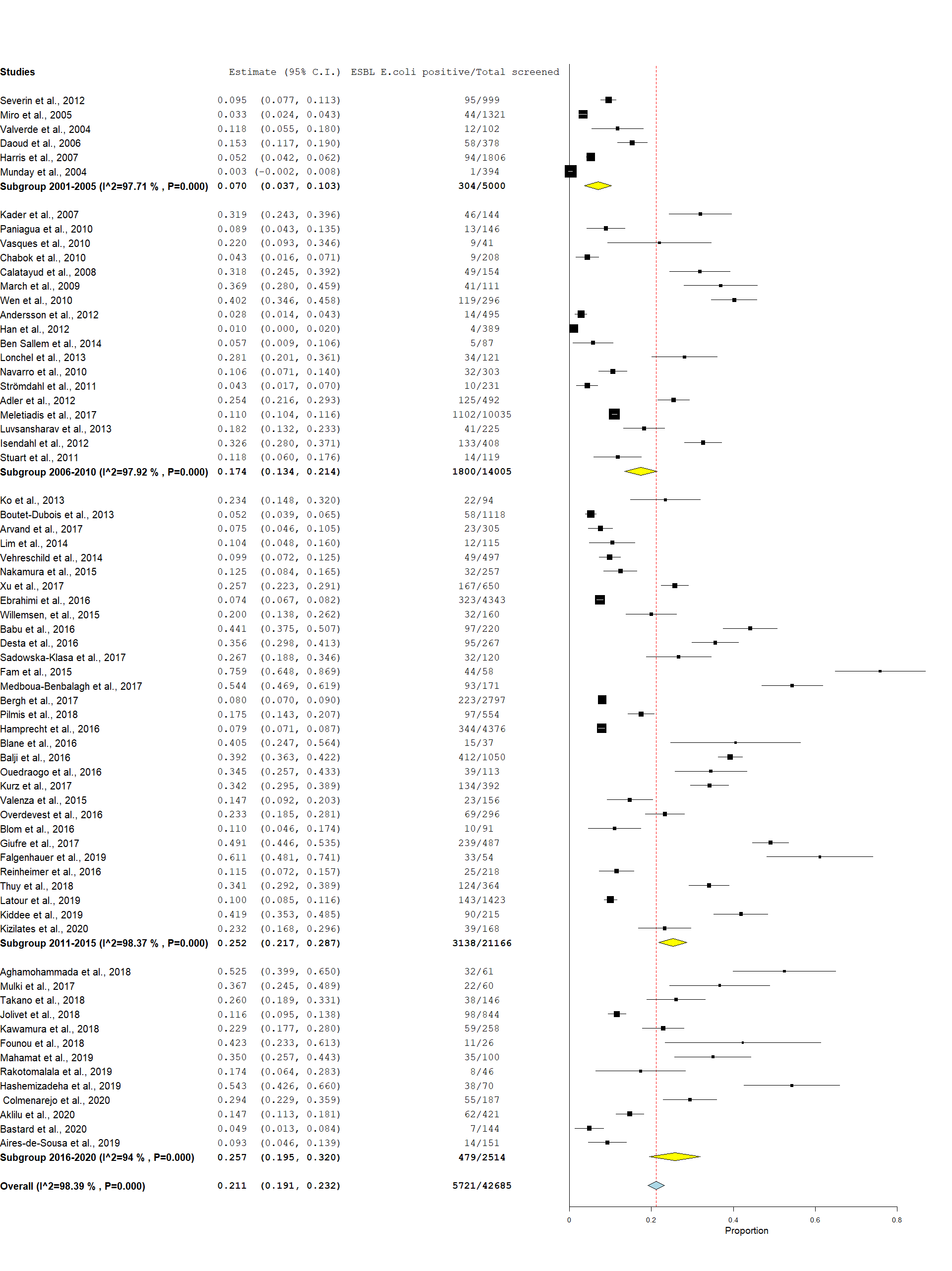


**Figure S5:** The global trend in the prevalence of human faecal ESBL *E. coli* carriage in the healthcare setting by subgrouping studies every five years of study period.


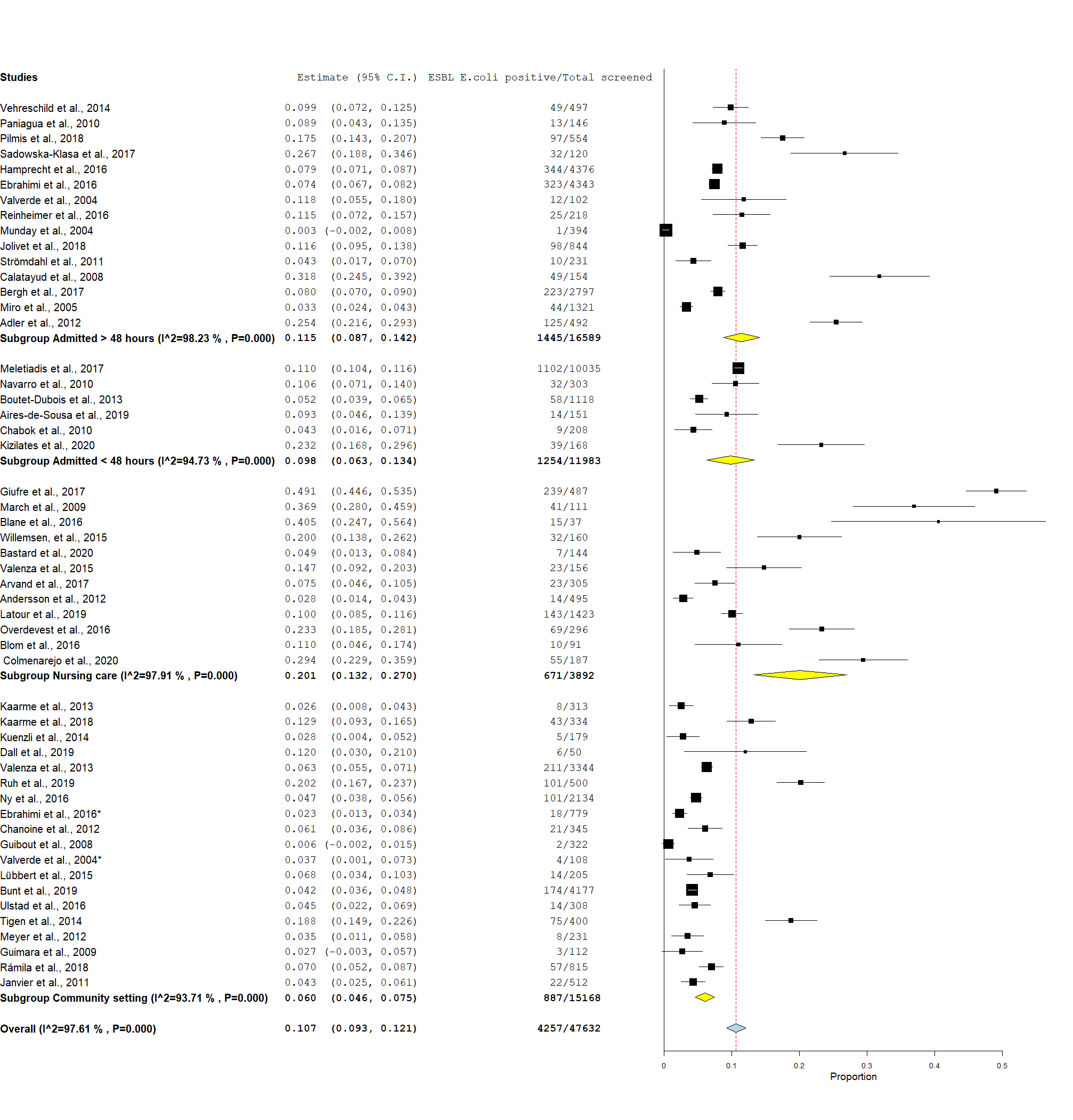
 **Figure S6:** The prevalence of human faecal ESBL *E. coli* carriage by amount (in time) of contact to healthcare settings in Europe.


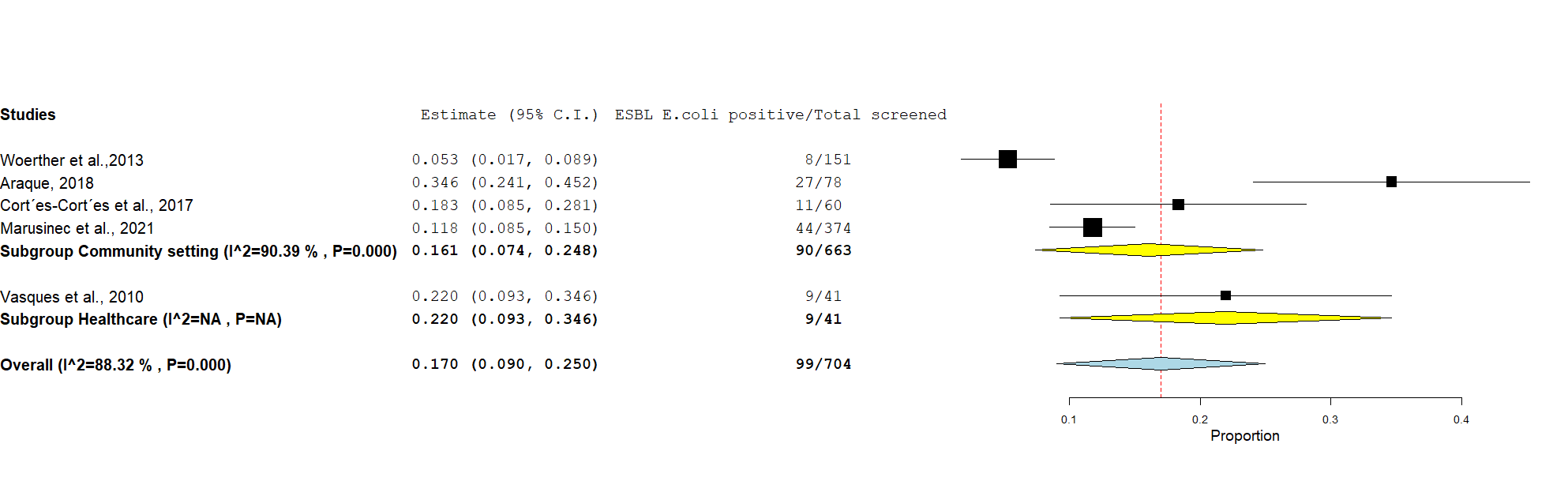


**Figure S7:** The prevalence of human faecal ESBL *E. coli* carriage in South Americas by study setting. Abbreviations: *E. coli, Escherichia coli*.


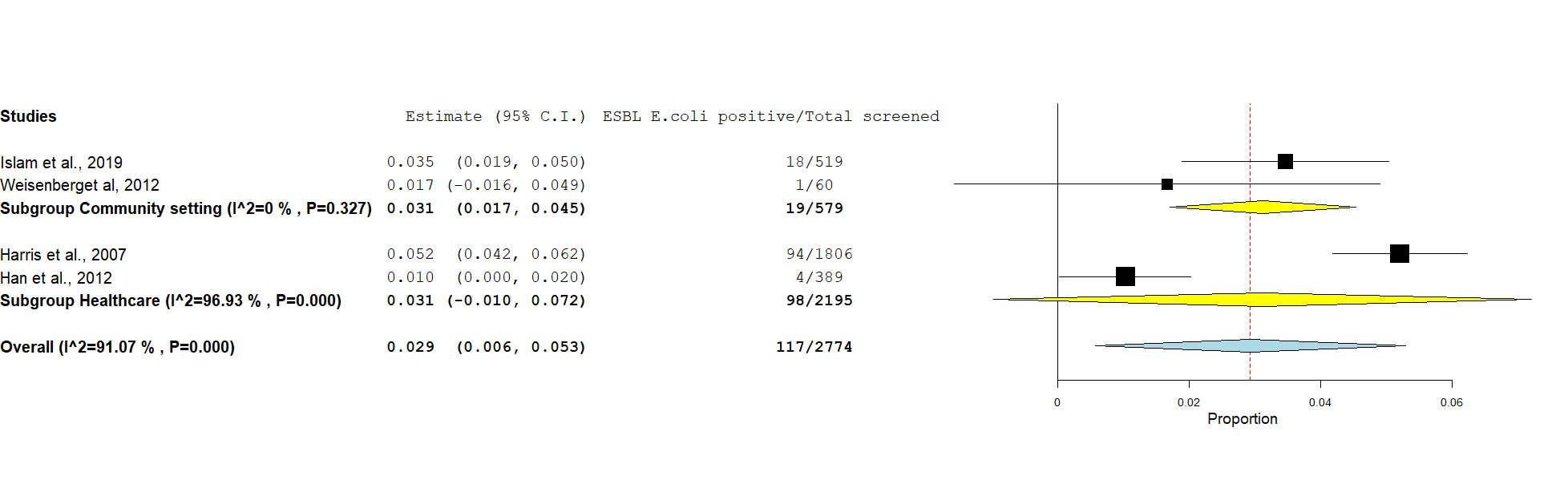


**Figure S8:** The prevalence of human faecal ESBL *E. coli* carriage in North Americas by study setting. Abbreviations: *E. coli, Escherichia coli*.


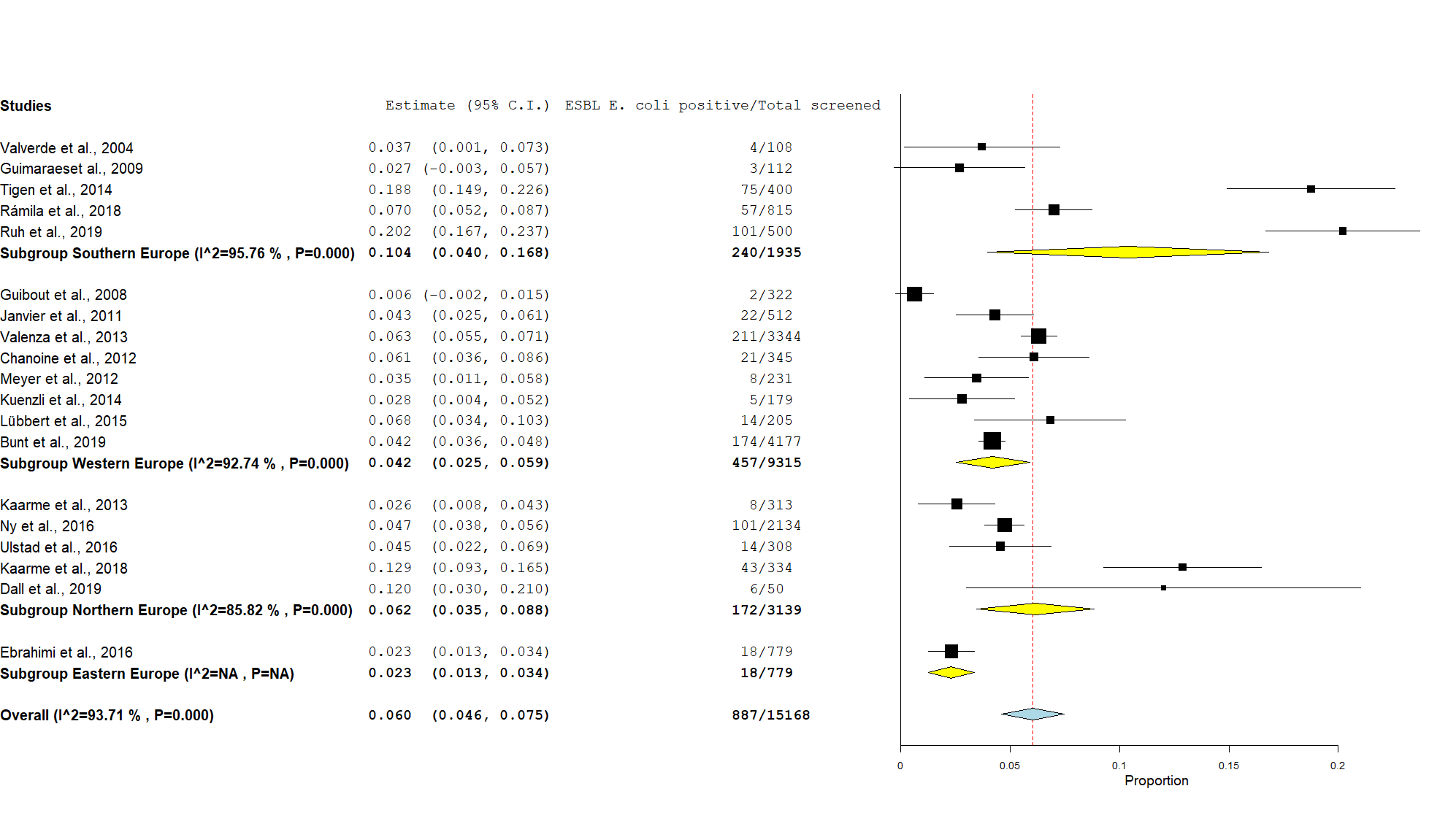


**Figure S9:** The prevalence of human faecal ESBL *E. coli* carriage in different sub-regions of Europe ^146^in the community setting. Abbreviations: *E. coli, Escherichia coli*


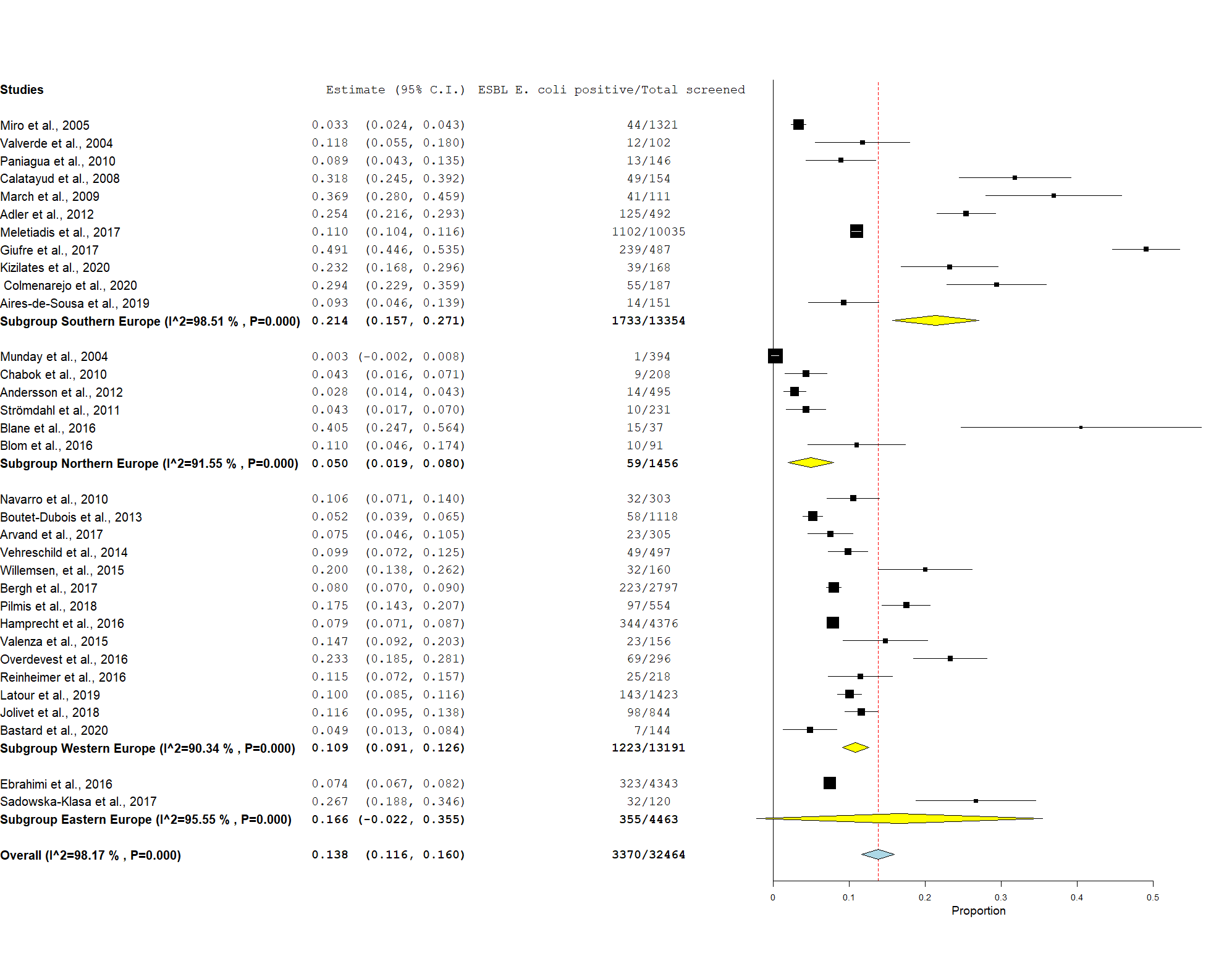


**Figure S10:** The prevalence of human faecal ESBL *E. coli* carriage in different sub-regions of Europe ^146^in the healthcare setting. Abbreviations: *E. coli, Escherichia coli*.


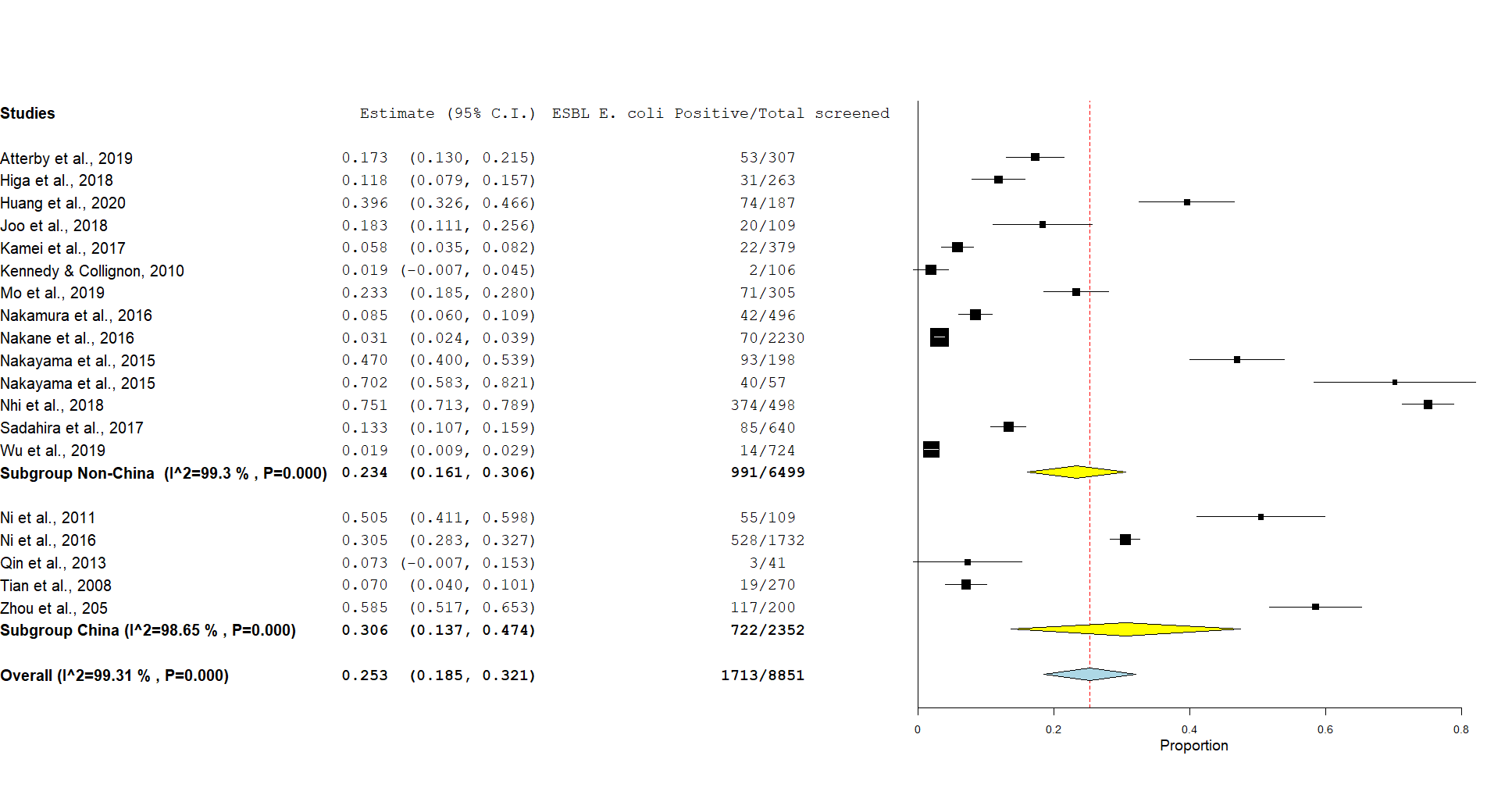


**Figure S11:** The prevalence of human faecal ESBL *E. coli* carriage in China versus the rest of West Pacific countries in the community setting. Abbreviations: *E. coli, Escherichia coli*.


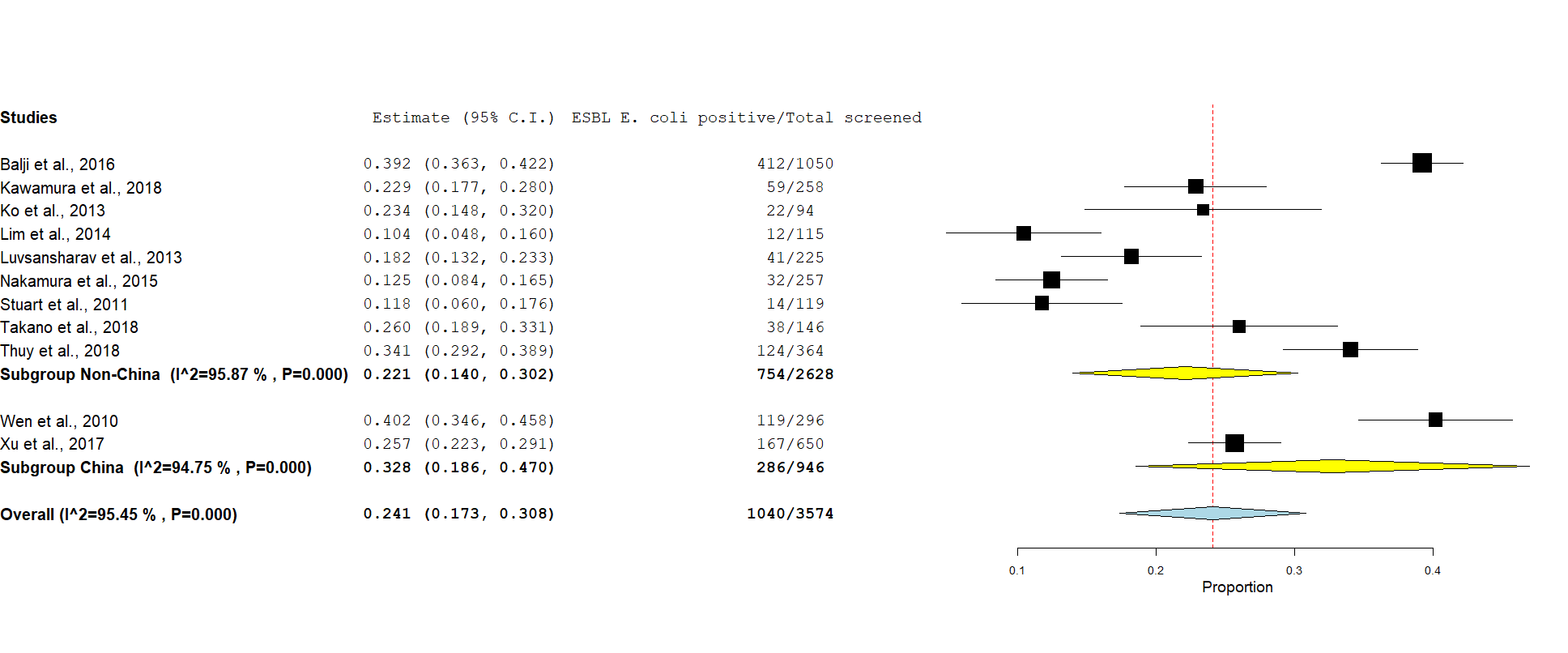


**Figure S12:** The prevalence of human faecal ESBL *E. coli* carriage in China versus the rest of West Pacific countries in the healthcare setting. Abbreviations: *E. coli, Escherichia coli*.

a)

b)

**Figure S13**: The prevalence of human faecal ESBL *E. coli* carriage in healthcare settings in different Sub-regions of Europe ^146^ (a). The figure below the bar graph (b) summarizes the prevalence of human faecal ESBL *E. coli* carriage by sub-region and study setting in Europe and Western Pacific regions. Abbreviations: *E. coli, Escherichia coli*. Note, non-China sub-region includes all West Pacific countries other than China.

**Figure S14**: Funnel plots of the included studies in community (a) and healthcare (b) settings. The dotted vertical lines pass through a value on x-axis that represent the global pooled prevalence for each study setting (17.6% for the community and 21.1% for healthcare settings). Lower prevalence with larger sample size in both plots was due to the presence of larger scale studies mainly from Europe which has a low ESBL E. coli carriage.
